# Diverse plant promoting bacterial species differentially improve tomato plant fitness under water stress

**DOI:** 10.3389/fpls.2023.1297090

**Published:** 2023-11-24

**Authors:** Elisa Zampieri, Elisabetta Franchi, Luca Giovannini, Francesca Brescia, Fabiano Sillo, Danilo Fusini, Ilaria Pietrini, Mauro Centritto, Raffaella Balestrini

**Affiliations:** ^1^ Institute for Sustainable Plant Protection, National Research Council of Italy, Turin, Italy; ^2^ Eni S.p.A., R&D Environmental & Biological Laboratories, San Donato Milanese, Italy

**Keywords:** *Solanum lycopersicum*, abiotic stress, PGPB, 16S, *in vitro* characterization, gene expression

## Abstract

**Introduction:**

Food crops are increasingly susceptible to the challenging impacts of climate change, encompassing both abiotic and biotic stresses, that cause yield losses. Root-associated microorganisms, including plant growth-promoting bacteria (PGPB), can improve plant growth as well as plant tolerance to environmental stresses. The aims of this work were to characterize bacteria isolated from soil and roots of tomato plants grown in open field.

**Methods:**

Biochemical and molecular analyses were used to evaluate the PGP potential of the considered strains on tomato plants in controlled conditions, also assessing their effects under a water deficit condition. The isolated strains were classified by 16S gene sequencing and exhibited typical features of PGPB, such as the release of siderophores, the production of proteases, and phosphorous solubilization. Inoculating tomato plants with eleven selected strains led to the identification of potentially interesting strains that increased shoot height and dry weight. Three strains were then selected for the experiment under water deficit in controlled conditions. The tomato plants were monitored from biometric and physiological point of view, and the effect of inoculation at molecular level was verified with a targeted RT-qPCR based approach on genes that play a role under water deficit condition.

**Results:**

Results revealed the PGP potential of different bacterial isolates in tomato plants, both in well-watered and stressed conditions. The used integrated approach allowed to obtain a broader picture of the plant status, from biometric, eco-physiological and molecular point of view. Gene expression analysis showed a different regulation of genes involved in pathways related to abscisic acid, osmoprotectant compounds and heat shock proteins, depending on the treatments.

**Discussion:**

Overall, results showed significant changes in tomato plants due to the bacterial inoculation, also under water deficit, that hold promise for future field applications of these bacterial strains, suggesting that a synergistic and complementary interaction between diverse PGPB is an important point to be considered for their exploitation.

## Introduction

The population across the world is increasing and it is estimated that it could reach 9.1 billion by 2050 (Pandey and Gupta, 2020). Agriculture is affected by different factors that cause yield reductions, such as drought, flooding, salinity, heat, cold, exposure to heavy metals as well as pathogen attacks (Pandey et al., 2017). In the last decade, an always more widespread practice to avoid the damage due to the climate change is the employment of plant biostimulants, such as soil microorganisms (*e.g.*, beneficial bacteria or mycorrhizal fungi) or natural substances, favoring plant nutrition and abiotic stress tolerance (Efthimiadou et al., 2020). Among biostimulants a promising group is represented by plant growth promoting bacteria (PGPB) that can produce hormones, antagonize pathogens, increase availability of nutrients as well as plant tolerance and resilience (Zampieri et al., 2022 and reference therein). Several studies have demonstrated the success of the inoculation with PGPB (Santos et al., 2019), suggesting that it might be a sustainable cultivation practice and healthy alternative to chemical fertilizers, antibiotics, herbicides, pesticides (Schlaeppi and Bulgarelli, 2015; Katsenios et al., 2021; Adedayo et al., 2022; Zampieri et al., 2022), also under abiotic stress conditions such as drought (Fadiji et al., 2022 and reference therein; Kour et al., 2022). This approach has been already tested on tomato (*Solanum lycopersicum* L.), *i.e.*, one of the major horticultural crops and source of vitamins A and C and carotenoids such as lycopene (Canene-Adams et al., 2005). Tomato is considered sensitive to water deficit, which causes a reduction in seed germination and development, in vegetative growth and reproduction (Nuruddin et al., 2003; Bartels and Sunkar, 2005; Rai et al., 2013). On average, the cultivation of tomatoes requires about 215 liters of water per kilogram (Mekonnen and Hoekstra, 2011). In the current scenario, characterized by long periods of drought, it has become crucial to develop strategies to obtain plants with enhanced drought tolerance.

A positive correlation between inoculation with PGPB and productivity has been highlighted in tomato plants (Kalam et al., 2020; Yavarian et al., 2021). Recently, Katsenios et al. (2021) showed that PGPB, added as solution in the soil close to industrial tomato (*cv.* Rio Grande), affected both the plant growth and metabolism, increasing the yield and improving the quality of the fruit in terms of carotenoids and lycopene. Positive effects of the tomato inoculation with PGPB have been also highlighted in other works, reporting an increase in yield (Gagné et al., 1993), lycopene, antioxidants, and potassium in fruits (Ordookhani et al., 2010), P levels in shoots (Hariprasad and Niranjana, 2009) as well as a more effective control of nematode and pathogen infections (Seleim et al., 2011; Shanmugam and Kanoujia, 2011; Almaghrabi et al., 2013). Inoculation with *Pseudomonas fluorescens* (SS5) in pot significantly increased tomato root and shoot weight, length, fruit yield *per* plant, and total fruit yield (Ahirwar et al., 2015). A field study demonstrated how PGPB belonging to *Pseudomonas* positively modulated tomato sugar production and the sweetness (Bona et al., 2017), while inoculation with *Kosakonia radicincitans* accelerated tomato flowering and ripening fruit and affected the ripened fruit amino acid, sugar and volatile composition (Berger et al., 2017). A *Bacillus* (isolate MT7), isolated from the rhizosphere of maize, showed the ability to colonize and to survive in the tomato roots after inoculation and to positive influence the plant growth in terms of root and shoot length, as well as fresh and dry biomass (Pathania et al., 2020). Additionally, there is evidence that inoculation with PGPB can improve tomato tolerance to abiotic stresses including water limitation (Bittencourt et al., 2023). Particularly, several papers have been dedicated to evaluate the tolerance of this relevant crop to both salt stress (Kissoudis et al., 2015; Van Oosten et al., 2018; Win et al., 2018; Yoo et al., 2019; Rojas-Solis et al., 2020; Ali et al., 2021; Hoffmann et al., 2021; Mellidou et al., 2021; Taj and Challabathula, 2021) and water limitation (Mayak et al., 2004; Iovieno et al., 2016; Brilli et al., 2019; Abbasi et al., 2020; Riva et al., 2021; Papadopoulou et al., 2022), both representing relevant threats for tomato productivity. According to the transcriptomic analysis conducted by Iovieno et al. (2016), in conditions of water deficit, tomato genes involved in processes such as photosynthesis, light harvesting, and the functioning of photosystems I and II, along with genes concerning cell proliferation and cell cycle, experienced down-regulation. Conversely, genes linked to the abscisic acid (ABA) pathway and stomatal movements showed an up-regulation (Iovieno et al., 2016). Brilli et al. (2019) verified that tomato plants pot-inoculated with *Pseudomonas chlororaphis* subsp*. aureofaciens* strain M71 showed an increased stress tolerance upon a mild water stress. Particularly, the M71 strain affected ABA level in leaves, resulting in the regulation of stomatal closure and a subsequent improving of water use efficiency (Brilli et al., 2019). In addition, the inoculation with M71 also affected proline content and antioxidant activity (Brilli et al., 2019). An improvement in water deficit tolerance was also reported by Mayak et al. (2004), in tomato plants inoculated with *Achromobacter piechaudii* ARV8 that increased seedling dried weight under transient water stress and aided the recovery upon rewatering. Inoculation of Tomato cv. Rio Grande with two *Streptomyces* isolates (IT25 and C-2012) allowed to reduce the weight fruit decrease under drought (Abbasi et al., 2020). Based on the results of a comprehensive greenhouse experiment in which tomato plants were exposed to either full irrigation or severe water deficit conditions in the presence of PGPB, the impact of the bacteria on plant productivity was assessed (Riva et al., 2021). Particularly, some strains were able to mitigate the stress and to improve the water use efficiency, and to increase the number of productive plants (Riva et al., 2021). The experiment carried out by Papadopoulou et al. (2022) demonstrated how *Pseudomonas putida* SAESo11 was able to mitigate the negative effects of drought in tomato plants and to increase H_2_O_2_ content and malondialdehyde levels. These last findings allowed to impose the plants in a primed status, able to reduce the injury due to drought and to respond strongly to the stress (Papadopoulou et al., 2022).

In the context of the application of PGPB in agriculture, it has been proposed that using consortia of PGPB instead of a single strain can significantly enhance the PGP mechanisms (Pandey et al., 2012; Mondal et al., 2020). For example, the use of two PGPB with complementary mechanisms of action can contribute to a comprehensive strategy that promotes growth and health aspects at the same time, due to the specialized functions of the used different bacteria (Mondal et al., 2020). The present study focused on the comprehensive characterization, both at the biochemical and molecular levels, of different bacterial strains isolated from the soil as well as from the roots of tomato plants grown in field. The objective was dual: firstly, to assess their efficacy on tomato plants in pot experiments under controlled, well-watered conditions, and secondly, to examine their performance in scenarios presenting stressors such as water deficits. Bacteria were assessed as single inoculum or as combinations, and outcomes were compared. This approach aimed at harnessing the synergistic potential of bacterial combinations in enhancing the tolerance of tomato plants to water deficit conditions.

## Materials and methods

### Isolation and characterization of bacteria from soil and roots

Bacteria were isolated from soil and roots collected during a sampling campaign at the ‘Azienda Pantanello’ (Basilicata, Southern Italy) in the frame of an in-field experiment to assess the effect of different water regimes on two tomato genotypes (Sillo et al., 2022). To isolate bacteria from soil, two approaches were used: a) enrichment and then selection; b) selection without enrichment. For approach (a), approximately 2 g of soil was added to 100 mL of Luria Bertani (LB) maximum medium in a 250 mL conical flask. The flasks were incubated at 30°C under stirring and after two days, 1 mL of the suspensions thus obtained were added to 100 mL of fresh medium (dilution 1:100) in two new 250 mL flasks. After two days at 30°C, serial dilutions were prepared and seeded on LB agar plates. After 48 hours at 30°C, some colonies were visualized. As expected, following the enrichment, the number of visualized phenotypes was limited. After sticking on fresh plates, the colonies were cleaned to obtain 14 pure colonies.

Instead, for approach (b), approximately 2 g of soil was added in 100 mL of minimum saline soil (KH_2_PO_4_ 1.5 g/L, NaHPO_4_ 0.5 g/L, NH_4_Cl 1 g/L, NaCl 0.1 g/L). The samples were incubated at 30°C with stirring for 16 hours. Serial dilutions 10^-5^, 10^-6^ and 10^-7^ were then prepared in sterile water, and 100 μL of these dilutions were distributed on R2A agar (Merck^®^) plates incubated at 30°C for five days. The R2A medium, less rich than the classic LB, allows a broad spectrum of bacteria to grow without the fast-growing bacteria suppressing the slow-growing species.

Several colonies with different phenotypes were visualized and then sticked onto fresh R2A plates. After the colonies were cleaned up to obtain 13 pure colonies from soil of tomatoes L (*cv.* Contact F1, called Lungo) and T (*cv*. Impact F1 called Tondo), irrigated with 100% of the estimated crop evapotranspiration (water regime R1) and 10 colonies from the soil of the same tomato genotypes (L and T) irrigated with 75% of the control treatment (water regime R2). Considering the two approaches, a total of 37 single strains were obtained from soil samples.

To isolate bacterial endophytes from plant roots, all root samples harvested in Sillo et al., 2022 (R1-L, R1-T, R2-L and R2-T) were kept separated (approach b). The soil around the roots was removed by repeatedly rinsing with tap water. Then, to sterilize the root surface, they were treated with 70% EtOH for 5 min, with NaClO for 2 min and again with 70% EtOH for another 5 min. After, they were thoroughly rinsed three times with sterile H_2_O. The roots, finely chopped with a sterile scalpel, were placed in sterile flasks containing TYEG (Trypticase Yeast Extract Glucose) medium and incubated for 16 hours at 30°C. Serial dilutions (10^-4^, 10^-6^, 10^-8^) of the obtained suspension were prepared, and 100 µL of each dilution (in triplicate) were spread on R2A agar (Merck^®^) plates. Several colonies appeared after 4-5 days, and after repeated streaking, we obtained 25 pure colonies from R1 root samples and 22 pure colonies from R2 root samples. A total of 47 strains were obtained from root samples. All isolated strains from soils and roots (84 strains) were used for DNA extraction and taxonomic classification as already described in Franchi et al. (2018).

### *In vitro* estimation of PGP activities

A BLASTn analysis was performed using the 16S rRNA sequences, obtained from the 84 isolates, and revealed the presence of several strains with Biohazard level 2 (according to three different databases accessed on 30 November 2022: “Classification of Prokaryotes—Bacteria and Archaea—into Risk Groups”, TRBA 466; www.baua.de/abas; Leibniz Institute DSMZ, German Collection of Microorganisms and Cell Cultures GmbH https://www.dsmz.de/; BCCM: Belgian Coordinated Collections of Microorganisms https://bccm.belspo.be/). Thus, only the 34 bacterial isolates classified with Biohazard Level 1 were subjected to a series of *in vitro* assays to assess their plant growth-promoting potential. The ability to solubilize inorganic phosphate (iP) was determined by culturing the strains in NBRIP (National Botanic Research Institute’s Phosphate) following the Nautiyal protocol (Nautiyal, 1999). The production of exopolysaccharides (EPS) was estimated using a modified Weaver mineral medium enriched with sucrose (Santaella et al., 2008). The production of indol-3-acetic acid (IAA) auxin was estimated following the method proposed by Shahab et al., 2009, while the ability to produce siderophore molecules was determined as described by Milagres et al. (1999). Proteolytic activity was determined as described by Nielsen and Sørensen, 1997. Ammonia production by growing strains in peptone water (5 g L^−1^ peptone and 5% NaCl, pH 7.2), according to the method of Kifle and Laing (2015). The isolated strains were also tested for their ability to form biofilms *in vitro* by inoculating them in glass tubes with 7 mL of LB medium. The tubes were incubated at 30°C for seven days without shaking. The formation of a visible layer (biofilm) at the interface between the culture medium and air indicates a potential ability to produce biofilm *in vivo*. The presence of the gene *nifH* encoding the nitrogenase reductase subunit and the widest marker gene used to identify nitrogen-fixing bacteria was looked for to determine the potential nitrogen-fixing capacity. The PCR to detect the presence of the *nifH* gene was performed with the two following degenerated primer pairs: 1) nifH-fwA (5’-GCIWTYTAYGGIAARGGIGG-3’); nifH-rvA (5’-GCCATCATYTCICCIGA-3’) and 2) nifH-fwA (5’-GCIWTYTAYGGIAARGGIGG-3’); nifH-rvB (5’-GCRTAIABNGCCATCATYTC-3’) using the following program: 94°C for 4’, repeated two times 94°C for 30’’, 65°C for 30’’, 72°C for 30’’, repeated two times 94°C for 30’’, 63°C for 30’’, 72°C for 30’’, repeated two times 94°C for 30’’, 61°C for 30’’, 72°C for 30’’, repeated two times 94°C for 30’’, 59°C for 30’’, 72°C for 30’’, repeated two times 94°C for 30’’, 57°C for 30’’, 72°C for 30’’, repeated 25 times 94°C for 30’’, 55°C for 30’’, 72°C for 30’’, 72°C for 2’. To detect the ability to produce the enzyme 1-aminocyclopropane-1-carboxylic acid (ACC) deaminase, the presence of the gene encoding acdS was also searched for, and the two following degenerated primer pairs were used: 1) acdS-fwA (5’-ATCGGCGGCATCCAGWSNAAYCANAC-3’); acdS-rvA (5’-GTGCATCGACTTGCCCTCRTANACNGGRT-3’) and 2) acdS-fwA (5’-ATCGGCGGCATCCAGWSNAAYCANAC-3’); acdS-rvB (5’-GGCACGCCGCCCARRTGNRCRTA-3’) using the following program: 94°C for 4’, repeated two times 94°C for 30’’, 66°C for 30’’, 72°C for 30’’, repeated two times 94°C for 30’’, 64°C for 30’’, 72°C for 30’’, repeated two times 94°C for 30’’, 62°C for 30’’, 72°C for 30’’, repeated two times 94°C for 30’’, 60°C for 30’’, 72°C for 30’’, repeated 25 times 94°C for 30’’, 58°C for 30’’, 72°C for 30’’, 72°C for 2’. For this purpose, we aligned, with the software Unipro UGENE, about 80 protein sequences (encoded by the *nifH* and *acdS* genes) from different soil microorganisms belonging above all to Actinobacteria, Firmicutes and Proteobacteria, identifying the most conserved areas and then drawing the degenerated primers on these sequences.

### Preparation of the PGPB inocula

Out of 34 obtained bacterial strains, eleven strains showing the greatest number of properties and, therefore, the most significant potential for promoting plant growth were selected for the *in vivo* tests (**Table 1**). For the preparation of the inocula, the strains were grown in LB medium for 48 h. After this step, the cell pellets obtained by centrifugation (9000 rpm, 20’) were resuspended with a solution composed of 1% sodium glutamate and 7% sucrose. The suspension was divided into small aliquots containing about 10^11^ CFU. The aliquots were frozen for 16 hours and then freeze-dried for storage until use.

**Table 1:** Phenotypic traits of selected inocula for the greenhouse experiment. In the table are reported the code of the inocula (SMV), the phenotypic traits such as Indole Acetic Acid production (IAA), inorganic Phosphate solubilization (iP), ammonia production (NH_3_), extracellular polysaccharides production (EPS), biofilm production (Biofilm), proteases production (Proteases), expression of nitrogenase iron protein 2 (*nifH2*), expression of 1-AminoCyclopropane-1-Carboxylate (ACC) deaminase structure gene (*acdS*) and the score.

SMV	origin	closest relative	Family	IAA	Siderophores	iP Solubil.	NH _3_	EPS	Biofilm	Proteases	*nifH2*	*acdS*	score
502	Soil	*Bacillus halotolerans*	Bacillaceae	─	─	+	+	─	+	+	─	─	4
504		*Rhodococcus erythropolis*	Nocardiaceae	+	─	─	+	+	+	─	+	─	5
509		*Leucobacter chromiireducens*	Microbacteriaceae	+	─	─	+	+	+	─	─	─	4
510		*Pseudochrobactrum saccharolyticum*	Brucellaceae	─	─	+	+	+	+	─	─	─	4
513		*Rothia amarae*	Micrococcaceae	+	─	─	+	─	+	+	─	─	4
514		*Bacillus butanolivorans*	Bacillaceae	─	─	─	+	─	+	+	+	─	4
515		*Peribacillus simplex*	Bacillaceae	+	─	─	+	─	─	+	+	─	4
518	Roots	*Sphingobacterium canadense*	Sphingobacteriaceae	─	+	+	+	+	─	─	─	─	3
522		*Variovorax boronicumulans*	Comamonadaceae	─	─	─		+	+	+	─	+	4
528		*Variovorax paradoxus*	Comamonadaceae	+	─	─	+	+	─	─	─	─	3
531		*Brevibacillus formosus*	Paenibacillaceae	+	+	─		─	+	─	+	─	3

Accession numbers: 502: OP364100, 504: OP364101, 509: OP364102, 510: OP364103, 513: OP364104, 514: OP364105, 515: OP364106, 518: OP364107, 522: OP364108, 528: OP364109, 531: OP364110.

### Seed sterilization and plant growth

In March 2022, San Marzano tomato seeds (La Semiorto Sementi Srl, Italy) were sterilized in 2.5% v/v sodium hypochlorite for 20 min, then they were rinsed five times and placed in Petri dishes on watered sterile paper. The Petri dishes were incubated at 25°C in a growth chamber for two days at dark and for other three days at light. After these five days, 62 tomato seedlings were transferred in a greenhouse to pots (0.7 L) containing quartz sand, previously sterilized at 180°C for 3h and watered with tap water. The plants were watered with tap water till a month from the transplanting, then they were watered two times a week with tap water and once a week with Hoagland solution (Hoagland and Arnon, 1950) at half concentration and grown set at a temperature of 24 ± 2°C, following the natural photoperiod.

#### Experiment #1

##### Inoculation with PGPB

After twenty-one days from the transplanting, the plants were inoculated with plant growth promoting bacteria, previously isolated and characterized (see above). For each strain five biological replicates were considered together with seven biological replicates for the uninoculated control. All the eleven different strains reported in **Table 1** were used; the lyophilized bacteria contained in 2 mL tubes were resuspended with 1.5 mL of sterile water and vigorously vortexed. The inoculation was performed by pipetting the solution in the pot at 1-2 cm of depth, tilting the tips of 45°, to reach the root apparatus. After the inoculation the pot surface was covered with coconut fiber to avoid the alga development. The plants were randomized each week. The inoculation was verified by a molecular approach based on the amplification of bacterial DNA in roots of inoculated plants, using primers for both (Ventura and Zink, 2002; Melničáková et al., 2013) the bacteria isolated from soil (509 and 510) and roots (518) (**Supplementary Material File**).

##### Morphological and physiological measurements

From 26/04/22 until 13/06/22 the plants were monitored two times a week to measure San Marzano height, stem diameter, and number of leaves. Height was measured from the shoot base to the last apical leaf, the diameter was measured with a digital caliper right under the first pair of leaves. At the same time, the portable chlorophyll meter SPAD 502 (CCM-200; Opti-Sciences) was used for the estimation of leaf chlorophyll content, one completely expanded leaf per plant was measured. On June 22, plants were removed from pots, submerged in tap water to remove the sand/coconut fiber from the root system and the shoot and root fresh weight measured. A small subsample of roots (around 700 mg) was stored at -20°C for subsequent molecular analyses. Then, roots and shoots were dried for five days at 50°C and weighed to measure the shoot and root dry mass.

#### Experiment #2

In October 2022, San Marzano seeds (La Semiorto Sementi Srl) were sterilized as described above and, after seven days, 160 seedlings were transferred to pots filled in with sterilized quartz sand (see above). The plants were grown at 25°C temperature, having 14 hours of light and 8 hours of dark. After nineteen days from the transplanting, the plants were inoculated with three selected strains showing the best results in Experiment #1, *i.e.*, strains 509, 510 and 518 separately, along with all their possible combinations, *i.e.*, 509 + 510, 509 + 518, 510 + 518, and 509 + 510 + 518. Twenty independent biological replicates (plants) were considered for each thesis, together with additional twenty uninoculated non treated control plants. The lyophilized bacteria, with the following CFU number: 1.08E^+11^ for 509, 1.73E^+11^ for 510, 9.60E^+10^ for 518 in 2 mL tubes, were resuspended with 1.5 mL of sterile water and vigorously vortexed, then the inoculation was performed using 750 µL of solution when the strain was used as pure. When the plants were inoculated with mixture of two strains, 375 µL of each bacterium was used, while when the mixture was of three strains, 250 µL of each bacterium was employed. After 16 days a second inoculation, following the same procedure of the first, was carried out. The plants were watered with tap water till a month and half from the transplanting, then they were watered two times a week with tap water and once a week with ½ Hoagland solution (Hoagland and Arnon, 1950). Out of 160 plants, 80 were used as controls (irrigated or WW) and maintained in a well-watered state (at pot capacity). The remaining 80 plants were subjected to a water limitation condition (water stress, WS) after one month and half from the transplanting, avoiding the pot irrigation. Before the stress beginning, biometric (height, number of leaves, stem diameter) and physiological (g_s_) measures were taken. Particularly, a LI-COR model LI-600 was used to verify the g_s_ and the stress level at the beginning and the end of the experiment. After ten days of stress the plants reached a moderate stress level and plants were sampled following this scheme: five replicates were dried for five days at 50°C and weighed to measure the shoot and root dry mass, while the remaining were frozen in liquid nitrogen and stored at -80°C.

### Quantitative gene expression analysis of leaves

Expression changes of the target transcripts were quantified on leaf sample by RT-qPCR. Three biological replicates for each condition (WW and WS) and for each inoculum (509, 510, 518 and each combination) were considered for the experiment. Total RNA was isolated by using a CTAB-based lysis buffer following the ‘pine tree method’ (Chang et al., 1993). The RNA pellet was resuspended in DEPC-treated water and quantity of the extracted RNA was determined by using a Nanodrop 1000 spectrophotometer (Thermo Fisher Scientific). RNA samples were then treated with TURBO™ DNase kit (Thermo Fisher Scientific), and genomic DNA contamination was checked before proceeding with cDNA synthesis by one-step RT-PCR using *SlCAC* specific primers of tomato (**Supplementary Table S1**). Total RNA for each sample was used to synthesize the cDNA, according to the SuperScript II Reverse Transcriptase^®^ (Invitrogen) procedure using random primers. Reactions were carried out in the ConnectTM Real-Time PCR Detection System (Bio-Rad Laboratories), and the SYBR Green method (Power SYBR Green PCR Master Mix; Biorad) was used to quantify the amplification results. Thermal cycling conditions were as follows: an initial denaturation phase at 95°C for 10 min, followed by 40 cycles at 95°C for 15 s and 60°C for 1 min. Specific annealing of primers was checked using dissociation kinetics performed at the end of each RT-qPCR run. The expression of tomato target transcripts was quantified after normalization to two established reference genes in leaves (*SlCAC*, a gene encoding a clathrin adaptor complexes medium subunit/endocytic pathway, and *Slexpressed*, coding an expressed sequence; Expósito-Rodríguez et al., 2008; Digilio et al., 2010). Gene expression data were calculated as expression ratios (relative quantity) using as control the data on uninoculated plants. The selected genes for gene expression studies were: a gene (*SlNCED1*) involved in the biosynthesis of the non-volatile isoprenoid ABA (Lopez-Raez et al., 2010), a gene coding for a protein kinase (*SlSnRK2*) with a role in abiotic stress response (Chitarra et al., 2016), a gene coding for a dehydrin (*SlTAS14*) (Chitarra et al., 2016) and a gene coding for a 1-aminocyclopropane-1-carboxylic acid oxidase (*SlACO4*) involved in the ethylene biosynthetic pathway (Porcel et al., 2014). In addition, a gene coding for a pyrroline-5-carboxylate synthetase (*SlP5CS*) (Iovieno et al., 2016) and two genes encoding for dehydration responsive element binding protein (*SlDREB1* and *SlDREB2*) were considered (Rai et al., 2019). A gene involved in volatile terpenes biosynthesis was also considered (*SlTPS12)* (Brilli et al., 2019), together with two genes coding for heat shock protein 20 (*SlHsp20*_I and II) and a gene coding for a NAC domain protein (*SlJa2*) (Iovieno et al., 2016). Gene-specific primers are listed in **Supplementary Table S1**.

### Statistical analyses

Statistical analyses were performed with R software (version 4.1.1). Data were transformed when necessary to fulfill ANOVA assumptions and one-way ANOVA was performed for the analysis of the data in Experiment #1, followed by Tukey’s HSD *post hoc* test. Two-way ANOVA was performed to assess inoculation and water deficit effects in Experiment #2 and when ANOVA indicated that for either condition (WW and WS) or bacteria inoculum (509, 510, 518, 509 + 510, 509 + 518, 510 + 518, 509 + 510 + 518) factors or their interaction was significant, mean separation was performed according to Tukey’s HSD test at a probability level of *p*  ≤ 0.05; ANOVA and Tukey’s HSD test were also used to analyze variability inside conditions and inoculations. The standard deviation (SD) or error (SE) of all means was calculated. A probability level of *p*  ≤ 0.05 was considered for all tests. Principal component analysis (PCA), performed using R software (version 4.1.1), was used to compare both the biometric and physiological data in the different considered inocula and in each condition (WW and WS). Radar plots were obtained by R software (version 4.1.1, commands “fmsb” and “ggradar”) on biometric parameters, to allow clearer visualization and compare differences between treatments. Concerning gene expression, statistical analyses were carried out using Relative Expression Software Tool REST^©^ 2009 v. 2.0.13 (Qiagen) (Pfaffl et al., 2002), considering 0.05 as significance of *p*-value. Only significant expression values were considered and visualized as heat maps by a custom R script (command “heatmap.2”). R software (version 4.1.1) was used to calculate the Pearson correlation matrix among the gene expression data.

## Results

### Isolation, identification and characterization of the bacterial strains

DNA amplification with primers for 16S allowed to identify the isolated bacterial strains, as reported in **Table 1**. Bacteria belong to Bacillaceae, Nocardiaceae, Microbacteriaceae, Brucellaceae, Micrococcaceae, Sphingobacteriaceae, Comamonadaceae, Paenibacillaceae families. Eleven bacterial strains isolated from tomato rhizosphere soil were characterized for various PGP traits (**Table 1**). Isolated strains showed different ability to produce specific biomolecules such as siderophores and to use inorganic phosphorous (iP) source. Strains 502, 513, 514, 515 and 522 were able to produce proteases. Strains 504, 509, 510, 518, 522 and 528 produced exopolysaccharides (EPSs). Strains 502, 510 and 518 were able to solubilize iP. Strains 504, 509, 513, 518, 528 and 531 produced IAA. All the strains with the exception of 515, 518 and 528 produced biofilms. Strains 518 and 531 are able to produce siderophores. Regarding NH_3_, all can release it, except for strains 522 and 531. Strains 504, 514, 515 and 531 coded for nitrogenase iron protein 2 (*nifH2*), while only strain 522 coded for ACC deaminase, based on amplification of *acdS*.

#### Morphological and physiological analysis under well-watered condition (Experiment #1)

**Figure 1:**
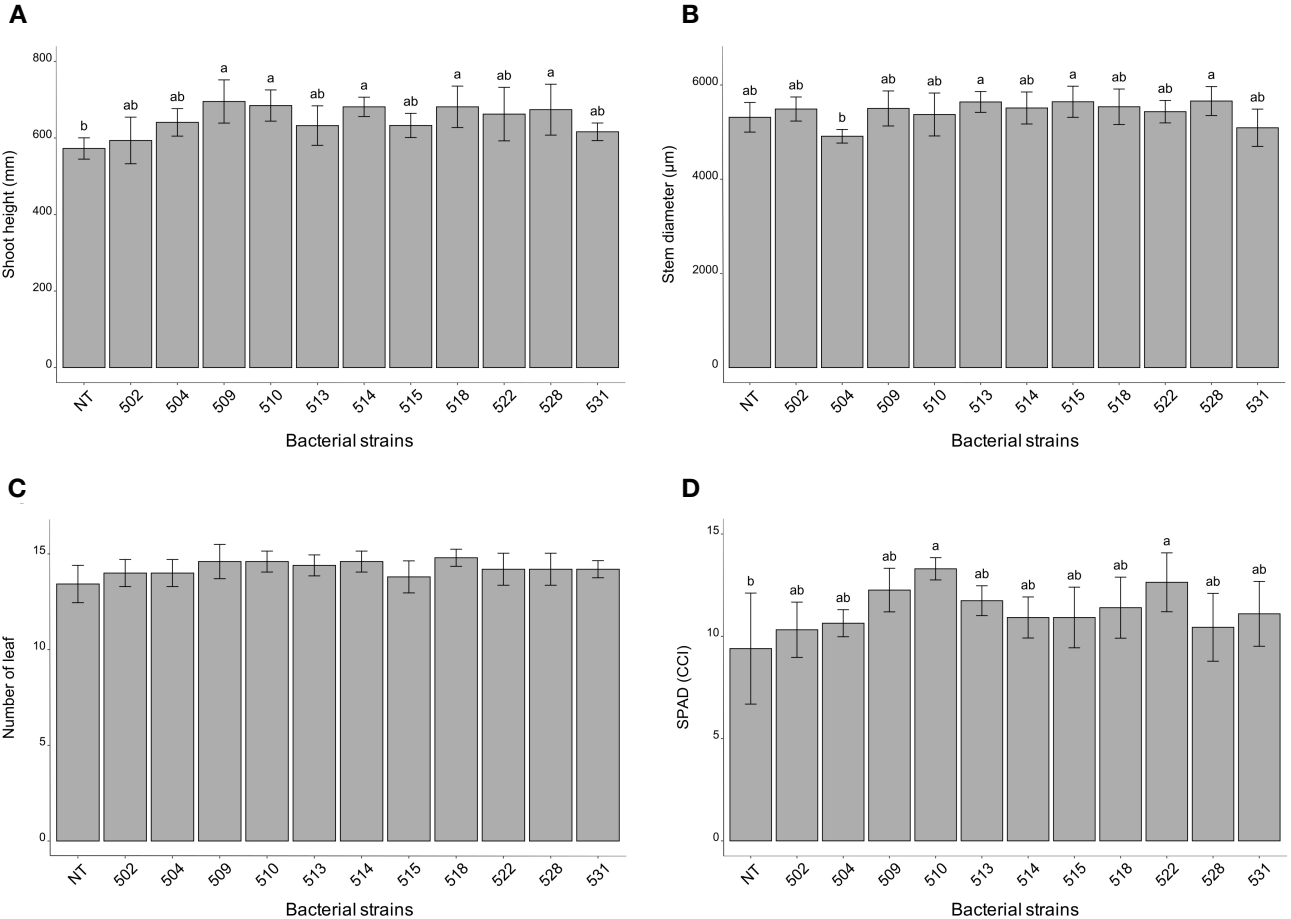
Histogram of plant height (mm) **(A)**, stem diameter (μm) **(B)** and number of leaves **(C)**, chlorophyll content (°SPAD) **(D)** taken on June 13 (last measurement day). Biometric data represent mean values ± SD, chlorophyll content as mean ± SE, in inoculated (n = 5) and non-inoculated (n = 7) plants. Letters are plotted on the base of Tukey’s test; different ones mean significant differences (*p* value <0.05).

**Figure 2:**
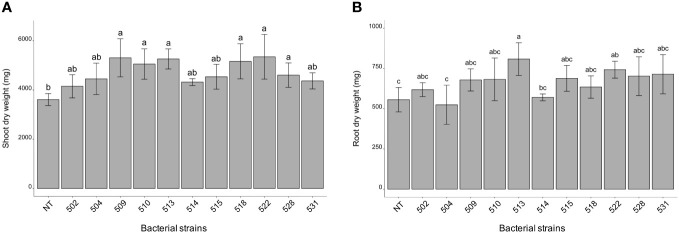
Shoot **(A)** root **(B)** dry weight of inoculated and non-inoculated (control) tomato cultivar (San Marzano). Data represent mean values ± SD in inoculated (n = 5) and non-inoculated (n = 7) plants. Letters are plotted on the base of Tukey’s test; different ones mean significant differences (*p* value <0.05).

The statistical analysis was carried out on the different morphological and physiological parameters, considering the measurements taken at the last day before the sampling. At the 13/06/2023, the plants inoculated with bacterial strains 509, 510, 514, 518, and 528 were statistically higher than the control plants (*p* 0.000475) (**Figure 1A** and **Supplementary Figure 1**). The stem diameter did not differ between inoculated and control plants, but the plants inoculated with strains 528, 515 and 513 were thicker than those inoculated with strain 504 (*p* 0.0137) (**Figure 1B**). From the point of view of leaf number there were not differences between inoculated and control plants (*p* > 0.05) (**Figure 1C**). The chlorophyll content, measured by SPAD meter, showed plants inoculated with strains 510 and 522 had more values than those of control ones (*p* 0.005) (**Figure 1D**). Considering the shoot dry weight, the heavier plants compared to the control were those treated with bacterial strains 509, 510, 513, 518, 522 and 528 (*p* 4.7e^-06^) (**Figure 2A**). Those were consistent with plant height except for the plants treated with strains 513 and 522. The latter showed heavier dried roots than the control (*p* 0.000125) (**Figure 2B**).

#### Morphological and physiological analysis under water deficit condition (Experiment #2)

**Figure 3:**
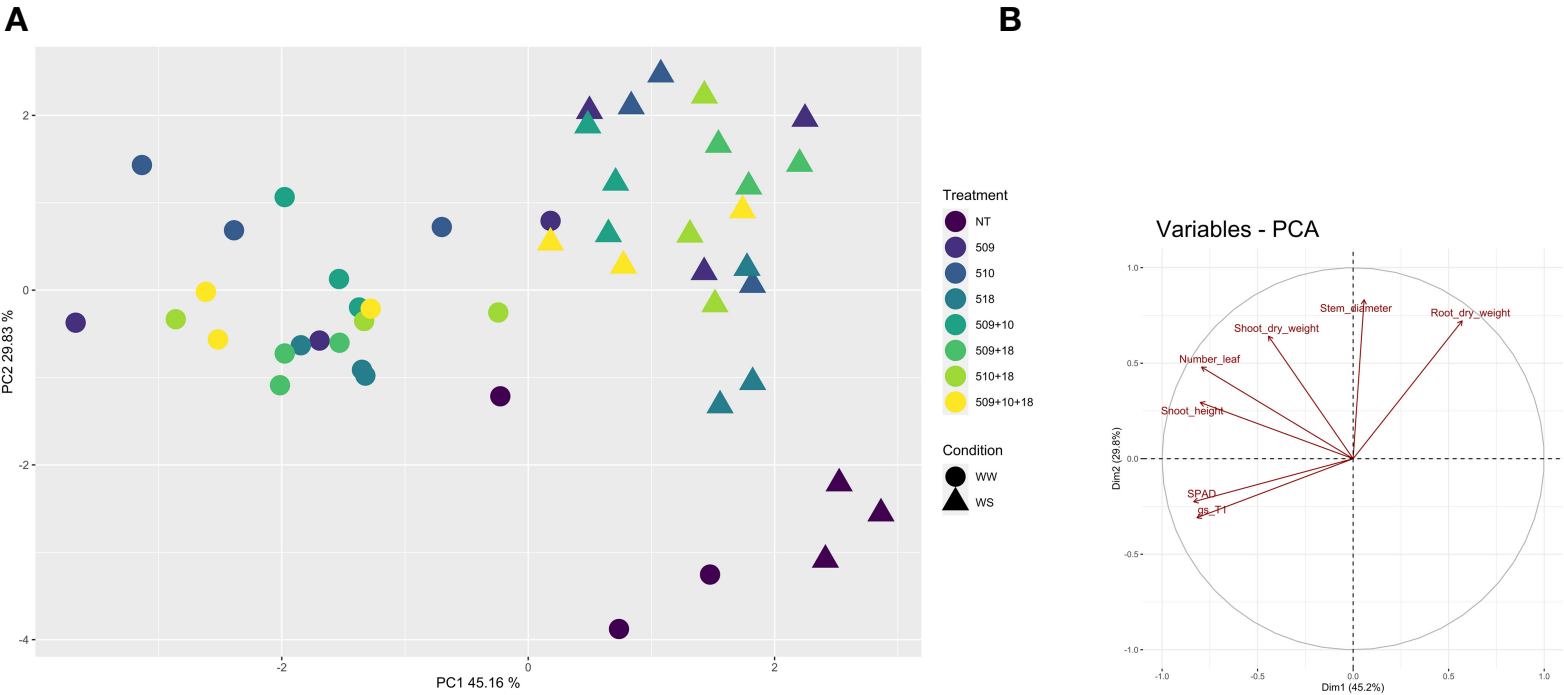
Principal component analysis of biometric and physiological parameters (shoot height, stem diameter, leaf number, root and shoot dry weight, chlorophyll content, and g_s_) performed with R (v 4.1.1) on the inoculated and non-inoculated plants in WW and WS condition. In **(A)**, Principal component analysis (PCA) of samples; in **(B)**, projection of variables, where angles are interpreted as correlations. The angle between two variable vectors represents the degree of correlation between them: adjacent (angle less than 90°) showed highly correlated variables, angle more than 90° showed uncorrelated ones.

**Figure 4:**
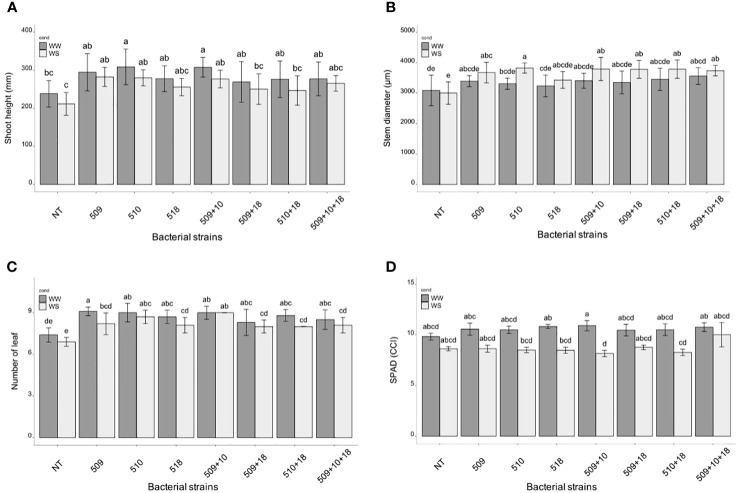
Histogram of plant height (mm) **(A)**, stem diameter (μm) **(B)** and number of leaves **(C)**, chlorophyll content (°SPAD) **(D)** in WW and WS condition. All biometric data are expressed as mean ± SD, while chlorophyll content as mean ± SE. Letters are plotted on the base of Tukey’s test; different ones mean significant differences (*p* value <0.05).

**Figure 5:**
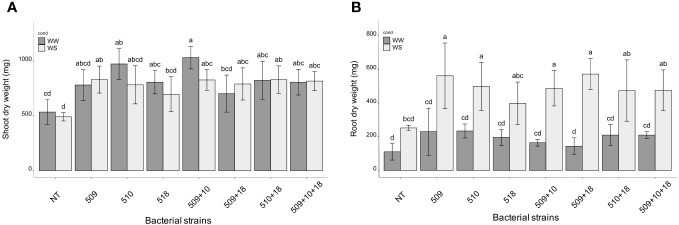
Shoot **(A)** and root **(B)** dry weight in WW and WS condition. All data are expressed as mean ± SD. Letters are plotted on the base of Tukey’s test; different ones mean significant differences (*p* value <0.05).

Plants were sampled once that the stressed ones showed g_s_ values under 0.01 (mol H_2_O m^-2^ s^-1^). The PCA, carried out on data of shoot height, stem diameter, leaf number, chlorophyll content, root/shoot dry weight and g_s_, recorded at the end of the stress period, showed that the control plants were separated from the inoculated ones and that there was a separation between WW and WS plants (**Figure 3**). This result has been also confirmed by the two-way ANOVA showing that condition factor resulted to significantly influence all biometric and physiological parameters with the exception of shoot dry weight (**Supplementary Tables S2** , **S3**). Conversely, inoculation factor significantly impacted all the considered parameters with the exception of CCI, while parameters were never significantly impacted by the interaction “condition x inoculation” (**Supplementary Tables S2**, **S3**). Considering the height, there were significant differences among treatments (*i.e.*, inoculation) (*p* 4.88e^-08^) and between conditions (*p* 0.00014). In particular, plants inoculated with strains 510 and 509 + 510 in well-watered and in stressed conditions were higher than the respective non-inoculated control plants. In addition, plants inoculated with strain 509 under stress were higher than stressed control ones (**Figure 4A**). Relating stem diameter, there were significant differences among treatments (*p* 1.64e^-08^) and between conditions (*p* 8.86e^-08^). All the inoculated plants under stress were thicker than control ones (**Figure 4B**). Concerning the number of leaves, there were significant differences among treatments (*p* < 2e^-16^) and between conditions (*p* 1.26e^-07^). In particular, all the inoculated plants under well-watered conditions had more leaves than control ones; under stress the plants inoculated with strains 510 and 509 + 510 had more leaves than stressed control ones (**Figure 4C**). The chlorophyll content measured by SPAD meter was significant between the conditions (*p* 2.42 e^-12^), the plants inoculated with strains 509 + 510 differed on the base of the presence of the stress condition (**Figure 4D**). Moving to dry weight of shoot, there were significant differences among treatments (*p* 9.03e^-8^): plants inoculated with strains 510 and 509 + 510 weighted more than control ones under well-watered conditions, while under stress plants inoculated with strains 509, 509 + 510, 509 + 518, 509 + 510, 509 + 510 + 518 had heavier shoots than stressed uninoculated controls (**Figure 5A**). From the root compartment, there were significant differences among treatments (*p* 0.001318) and between conditions (*p <*2.2e^-16^). In particular, well-watered inoculated plants were not different from the control ones, while under stress plants inoculated with strains 509, 510, 509 + 510 and 509 + 518 differed from control ones, having a more weight (**Figure 5B**).

The radar plots, performed for each biometric parameter, confirmed the findings of the PCA analysis. Specifically, they clearly separated non-inoculated plants from inoculated ones, a trend observable in both well-watered and stressed conditions (**Supplementary Figure 2**). Upon examining the data pertaining to plant height, it was observed that the strain 510 and the strain combination 509 + 510 were the most influential, followed by the strain 509. When considering stem diameter, all strains seemed to influence this parameter under stressed conditions. Furthermore, the strain 509 and the combination 509 + 510 had a notable impact on leaf number, followed by strain 510. Regarding shoot dry weight, the strains 509 + 510 and 510 were the dominant contributors. Lastly, under stressed conditions, the root dry weight was predominantly influenced by the strains 509 and 509 + 518 (**Supplementary Figure 2**).

### Gene expression

**Figure 6:**
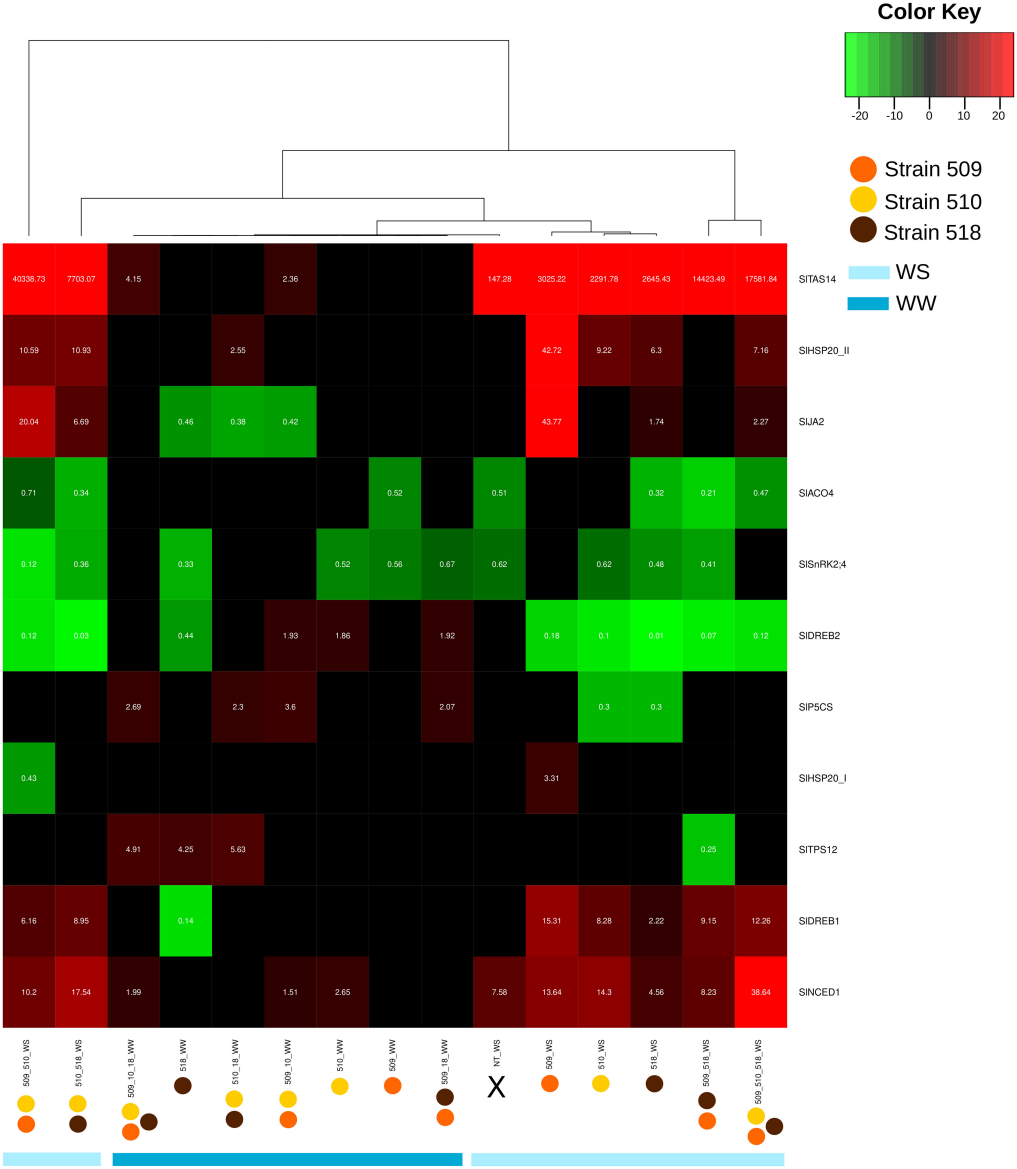
Heat map representation of the transcript level (as result of the fold change calculated following 2^−ΔΔCT^) to a hierarchical clustering in well-watered (WW) and under water stress (WS) inoculated and non-inoculated (NT) plants. Each column represents a treatment, while each row represents a gene. Expression levels are colored green for low intensities and red for high intensities (see scale at the top right corner). The black cells represent genes not significantly different from those of the untreated samples.

Leaf transcript levels of eleven genes potentially involved, on the basis of the literature data, in the stress response were evaluated in the inoculated plants, under stress and in well-watered conditions (**Supplementary Tables S4**, **S5**). **Figure 6** shows the heatmap representation of the significantly differently expressed genes, in the different conditions. The inoculation with strains 509 + 510 and with 518 in WS showed the greatest number of significantly regulated genes, while the inoculation with strain 509 in WW showed the most limited number of regulated genes. The attention has been focused on genes coding for proteins involved in plant response to stress, volatile terpene biosynthesis and hormonal pathways. Among the genes potentially involved in water deficit response, *SlNCED1* and *SlTAS14* code for a protein involved in the biosynthesis of the non-volatile isoprenoid abscisic acid (ABA; Qin and Zeevaart, 1999; Lopez-Raez et al., 2010) and a tomato dehydrin accumulating in presence of mannitol, ABA and salt (Sacco et al., 2013), respectively. Particularly, the transcriptional level of *SlNCED1*, coding for a 9-cis-epoxycarotenoid dioxygenase involved in ABA biosynthesis, increased in the well-watered plants inoculated with strains 510, 509 + 510 and with 509 + 510 + 518, and in all the plants under water stress condition such as the dehydrin gene *SlTAS14*. It is worth noting that this last was also up-regulated in well-watered plants inoculated with diverse bacterial combination, *i.e.*, strains 509 + 510 and 509 + 510 + 518. *SlTPS12*, coding for a terpene synthase (Falara et al., 2011), was up-regulated in well-watered plants inoculated with strains 518, 510 + 518, 509 + 510 + 518, while it was down-regulated in stressed plants inoculated with strains 509 + 518. Ethylene is a gaseous stress hormone that generally increased in the root systems of plants subjected to water limitation, causing the inhibition of root growth (Brunetti et al., 2021). *SlACO4*, coding for an ACC oxidase (ACO) putatively involved in the ethylene biosynthetic pathway (Porcel et al., 2014; Pattyn et al., 2021), was down-regulated in well-watered plants inoculated with 509 and in stressed plants inoculated with the strains 518, 509 + 510, 509 + 518, 510 + 518, 509 + 510 + 518 as well as in uninoculated stressed plants. *SlSnRK2;4*, coding for a serine/threonine-protein kinase (Sun et al., 2011), was down-regulated in well-watered plants inoculated with strains 509, 510, 518, 509 + 518, and in all the stressed plants with the exception of the plants inoculated with strains 509 and 509 + 510 + 518, where it was not significantly regulated. Plants stimulate the production and accumulation of osmoprotectant compounds (*e.g.*, amino acids, proteins, sugars) and osmolytes such as proline (Takahashi et al., 2020) under osmotic stress conditions. Among the tomato genes up-regulated in leaves of plants maintained in water deficit conditions and reported in Iovieno et al. (2016) also the gene *SlP5CS*, coding for a pyrroline-5-carboxylate synthetase involved in proline biosynthesis. In our study, this gene was up-regulated in plants inoculated with strains 509 + 510; 509 + 518; 510 + 518 and 509 + 510 + 518. Under water stress it was down-regulated in plants inoculated with strains 510 and 518. *SlDREB1*, coding for a dehydration responsive element binding protein previously found in ethylene mediated signaling pathway, transcription initiation and defense response (Rai et al., 2019), was down-regulated only in the well-watered plants inoculated with strain 518, while in all the stressed plants it was up-regulated with the exception of uninoculated plants. *SlDREB2*, coding for a dehydration responsive element binding protein 2 (Rai et al., 2019), was instead up-regulated in well-watered plants inoculated with strains 510, 509 + 510, and 509 + 518, while it was down-regulated in strain 518 inoculated plants and in all the stressed and inoculated plants. *SlHSP20_II*, coding for heat shock protein 20, previously reported as up-regulated in tomato plants subjected to water deficit (Iovieno et al., 2016), was up-regulated in all the stressed and inoculated plants with the exception of those inoculated with strains 509 + 518, where it was not significant regulated, and in well-watered plants inoculated with strains 510 + 518. The gene *SlHSP20_I* was up-regulated exclusively in stressed plants inoculated with strain 509 and it was on the contrary down-regulated in plants inoculated with strains 509 + 510. *SlJA2*, coding a NAC domain protein that was found to be up-regulated in tomato leaves upon a water deficit conditions and suggested to promote stomatal closure through induction of expression of the ABA biosynthetic gene *NCED1* (Iovieno et al., 2016), was down-regulated in well-watered plants inoculated with strains 518, 509 + 510, 510 + 518, while it was up-regulated in stressed plants inoculated with strains 509, 518, 509 + 510, 510 + 518, 509 + 510 + 518.

**Figure 7:**
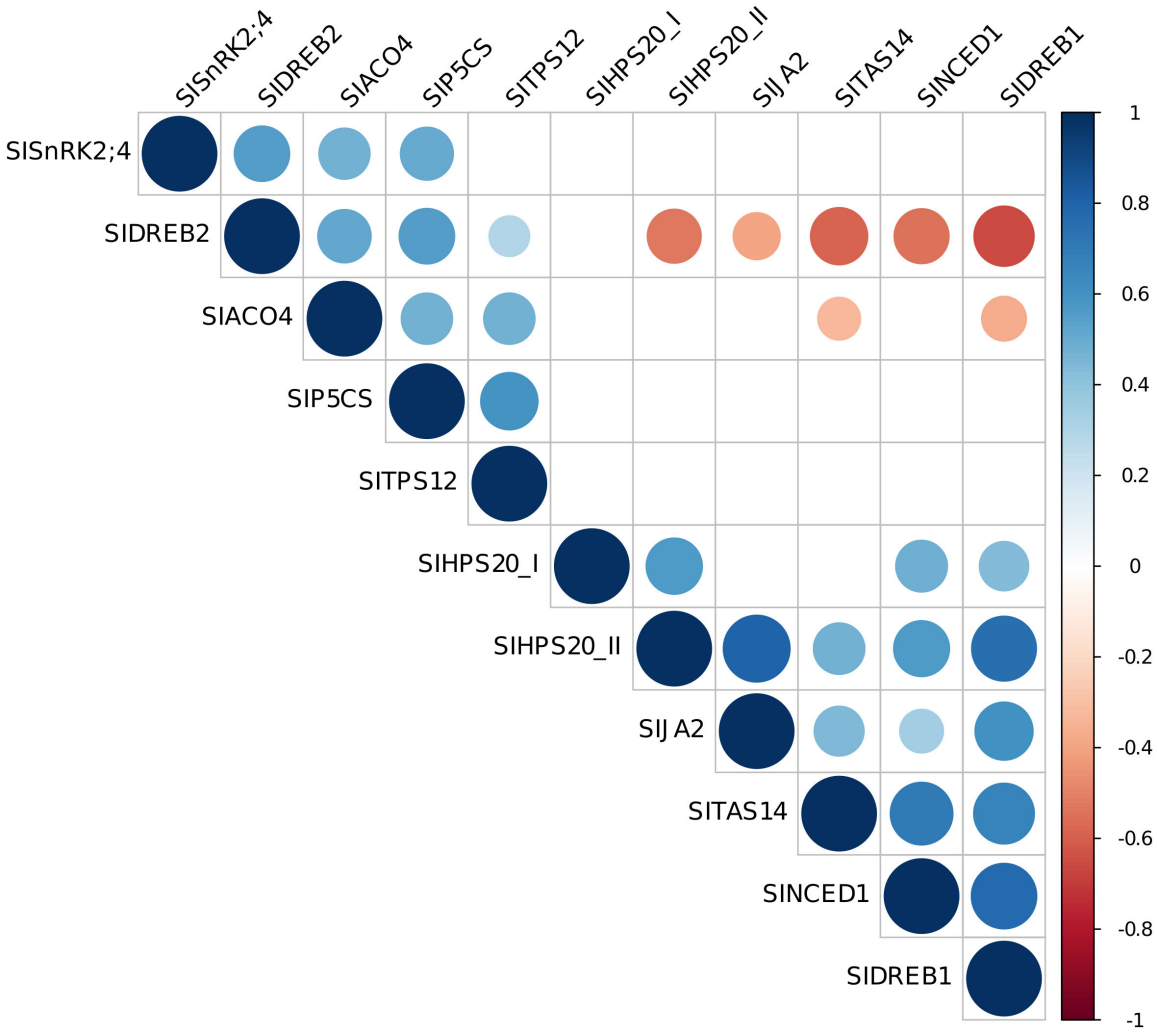
Correlation plot among gene expression data. Visualization of significant correlations between each pair of variables is shown. Stronger correlations are represented by darker colors and larger circles. Blue indicates positive correlation, while red indicates negative correlation.

On the basis of the correlation among gene expression data, it was possible to distinguish two groups: genes for which their expression is positively correlated among each other (*SlSnRK2;4*, *SlDREB2*, *SlACO4*, *SlP5CS*, *SlTPS12*), and genes (*SlHSP20_II*, *SlJA2*, *SlDREB1*, *SlNCED1*, *SlTAS14*) negatively correlated with *SlDREB2* and *SlACO4* (**Figure 7** and **Supplementary Figure 3**).

## Discussion

It is known that the PGPB can help in managing soil quality and other environmental factors associated to crop limited growth and yield (Yadav et al., 2020). PGPB can directly or indirectly act by mobilizing and solubilizing nutrients, synthetizing phytohormone and inducing systemic resistance leading to an increased plant growth (Pérez-Jaramillo et al., 2018). Here, the impact of bacterial strain inoculation has been evaluated on *S. lycopersicum*, also under water deficit, after the bacterial characterization. Particularly, a biochemical characterization of eleven bacterial strains isolated from the soil and roots of tomato plants growth in open field was carried out to assess their *in vitro* PGP potential. All these strains were used for tomato inoculation to verify the effect on plant growth under optimal water condition.

### Bacterial characterization and their impact on tomato growth

Among the characterized strains, it is worth noting the presence of *Bacillus halotelarans* that is a species harboring strains with growth promoting effects, as recently reported by Tsalgatidou et al. (2023), and *B. butanolivorans*, which is known to be able to induce drought tolerance in pepper plants (Kim et al., 2022). A *Peribacillus simplex* strain was isolated from soil of tomato cultivation and, interestingly, this species has shown the capacity to accumulate lead and to have a biosorption potential (Chamekh et al., 2021). Additionally, *Rhodococcus erythropolis*, here isolated and characterized, is known to grow on polluted soil, mainly in presence of toxic Cr^6+^ concentration (Trivedi et al., 2007). A species belonging to *Rothia* genus was found in this study. Inoculations with *Rothia* sp. demonstrated to alleviate the negative effects of pest infestation and to increase plant biomass and yield in infested plants (Bano and Muqarab, 2017). Two species belonging to *Variovorax* genus were also identified in the tomato roots, *i.e.*, *V. boronicumulans* and *V. paradoxus*, known to positively affect the plant growth, also in response to environmental stress and metal accumulation (Sun et al., 2018; Flores-Duarte et al., 2022).

Among the isolated strains, 509, 510, and 518 were further characterized under water deficit condition as they showed the most promising traits both *in vitro* and *in planta*. These strains were identified as *Leucobacter chromiireducens* (here called strain 509), *Pseudochrobactrum saccharolyticum* (here called strain 510) and *Sphingobacterium canadense* (here called strain 518), respectively. All these genera have already reported to present diverse PGP traits, such as phytohormone (IAA) and siderophore production for *Sphingobacterium* and *Pseudochrobactrum* in addition to ACC deaminase activity, and phosphorous solubilization in *Pseudochrobactrum* (Moon and Ali, 2022; Querejeta et al., 2022; Shi et al., 2022). The strains 509, 510 and 518 showed different PGP traits, such as the EPS and biofilm production and the capacity to release NH_3_. In addition, strain 509 showed the ability to produce IAA, while strains 510 and 518 were able to solubilize phosphate, and strain 518 showed ability to release siderophores.

Concerning strain 509, the capacity to produce IAA and release NH_3_ was in agreement with literature, while solubilization of iP was a feature that, at our knowledge, was exclusively detected in our strain. Strain 518, in agreement with the literature, produced siderophores and solubilized iP, but, differently from other strains of the same species, it was not able to produce IAA. On the other hand, characterization data on strain 510 allowed to associate unexpected features, such as the ammonia production and extracellular polysaccharides production, to this bacterial strain. These strains demonstrated to have a positive effect after inoculation *in planta*, increasing the shoot height and dried biomass at least in well-watered condition, while in water limitation the best inocula were strains 510 and the combination 509 + 510 that increased the shoot height, number of leaves and shoot dry weight. All the inoculated plants also showed an increase in root biomass under stress, in agreement with a previous work on tomato plants inoculated with PGPB (Mayak et al., 2004). A common pattern between strains 509 and 510, based on the capacity to produce biofilm, EPS and NH_3_, was observed. The synthesis of biofilm by bacteria is known to confer various benefits to PGPB. These advantages include enhanced tolerance to abiotic stress, facilitated plant interactions, increased protection against biotic stresses, and a more efficient nutrient acquisition. Consequently, these benefits can be extended to plants, potentially leading to increased yields (Schlaeppi and Bulgarelli, 2015; Ajijah et al., 2023). In addition, a recent review on *Pseudomonas* spp. highlighted as rhizosphere soil structure and water content under abiotic stresses were improved by bacterial production of EPSs (Zboralski and Filion, 2023).

### Impact of the bacterial inoculation on the expression of genes involved in stress responses and different hormonal pathways

To assess the molecular mechanisms involved in the plant response to different inocula in presence and absence of an abiotic stress, genes known to have a role in plants subjected to water deficit have been tested. Among them, genes related to plant response to stress and to the metabolism and signaling pathway of the plant hormone ABA, which is known to mediate adaptive responses to abiotic stresses (Yang et al., 2022), were assessed, including *SlNCED1*, *SlTAS14*, *SlSnRK2.4, SlTPS12, SlDREB1, SlDREB2*, and *SlJA2*. The variations in data between our study and previous literature might be attributed to the utilization of diverse plant genotypes and bacterial species, as well as differing growth and water stress conditions. This highlighted the importance of further exploration and understanding of the complex interactions played in these biological systems. Particularly, *SlNCED1*, coding for a key enzyme in the biosynthesis of ABA that is a plant hormone that responds rapidly to environmental changes (Barta and Loreto, 2006), was up-regulated in all stressed conditions, but also in the well-watered plants inoculated with single strains and combination of them. Brilli and colleagues observed the up-regulation of *SlNCED1* in stressed non-inoculated plants. On the other hand, Chitarra and colleagues identified an up-regulation of this gene in plants under water stress that were inoculated with the arbuscular mycorrhizal fungus *Funneliformis mosseae*, indicating a cooperative role in modulating ABA pathways shared by both beneficial organisms (Chitarra et al., 2016; Brilli et al., 2019). The activation of ABA pathway in the absence of stress signals may represent a form of biological priming, where the plant is prepared to respond more robustly if and when stress conditions occur. In our study, it was observed that *SlNCED1* was also up-regulated in well-watered plants inoculated with strains 510, 509 + 510, and 509 + 510 + 518, suggesting a potential priming effect, by acting on ABA regulation, probably linked mainly to strain 510. Notably, among the tested bacterial strains collected from soil, strain 510 showed an exclusive ability to solubilize iP. Recently, studies in rice have reported that genes associated with the ABA pathway, including *OsNCED2*, play a relevant role in modulating P homeostasis (Haider et al., 2023), and studies on *Arabidopsis ABI5* mutant showed that ABA positively regulate P acquisition through ABI5 (Zhang et al., 2022). The transcript level of the ABA-responsive dehydrin *SlTAS14* was also evaluated, being a well-known marker of drought stress response, able to play a role in the increase of solute quantities in the cells when an osmotic stress occurs (Muñoz-Mayor et al., 2012). This gene was clearly induced in tomato plants subjected to water stress, as previously observed in tomato plants during short and long water deficit periods, after a treatment with a natural biostimulant based on plant polyphenols (Hamedeh et al., 2022). It has been demonstrated that this gene enhances tolerance to both drought and salinity, improving the capacity to rapidly increase ABA after the perception of the stress (Muñoz-Mayor et al., 2012). In addition, Goñi et al. (2018) showed an up-regulation of this gene in Moneymaker tomato plants under drought and after the inoculation with *Ascophyllum nodosum*; the regulation changed in relation to the treatment, suggesting that the plants experienced different degrees of drought tolerance (Goñi et al., 2018). The up-regulation of *SlTAS14* in two inoculated (with strains 509 + 510 and 509 + 510 + 518) well-watered plants could instead be explained with the role of dehydrins in the plant growth under normal condition (Liu et al., 2017), or, alternatively, by a priming effect as observed for *SlNCED1*. A further gene involved in the ABA signaling investigated in our study is *SlSnRK2.4*, which is able to interact with *SlAREB1* and *SlAREB2*, *i.e.*, the major downstream transcription factors of ABA-dependent signaling pathway (Liu et al., 2021). In all the inoculated stressed plants, a down-regulation of this gene was observed, except in the cases of plants inoculated with either the strains 509 or the 509 + 510 + 518, where the gene expression remained similar to that of the control group. This could potentially be explained by the fact that the signaling pathway was orchestrated by other genes, such as *SlPYLs* and *SlPP2Cs*, which are recognized for their roles in drought response mechanisms, as already observed (Chitarra et al., 2016). Interestingly, in sugarcane roots, the inoculation with *Gluconacetobacter diazotrophicus* in presence of a water deficit condition determined the inhibition of ABA biosynthesis, signaling and response (down-regulating genes like *SnRK2*, *DREB, NCED*), in comparison with non-inoculated sugarcane in the same abiotic conditions (Vargas et al., 2014). The methyl-erythritol phosphate (MEP) pathway has been reported to be strictly associated to those related to monoterpene biosynthesis (Brilli et al., 2019). Among the terpene synthase genes, *SlTPS12* was up-regulated by some bacterial strains and combinations as previously reported by Brilli et al. (2019), which showed its up-regulation in tomato under optimal water conditions. This result can suggest that the bacteria may activate defense mechanisms against abiotic and biotic stresses (Loreto and Schnitzler, 2010), supporting the hypothesis that a priming effect might occur. By contrast, the water stress probably turned off the gene, even in presence of bacterial strains, as already observed (Brilli et al., 2019).

Genes belonging to the DREB1 family have been documented to be directly involved in tolerance to cold temperatures, drought, and salt (Nakashima and Yamaguchi-Shinozaki, 2006). Particularly, drought, exogenous ABA, salt and trehalose are also known to regulate the expression of *SlDREB1* (Jiang et al., 2017; Rai et al., 2019; Yu et al., 2019). Moreover, it is recognized that *SlDREB2* gene plays an important role in orchestrating the expression of stress-resistant and functional genes, improving tolerance to water deficit in plants (Tao et al., 2022). In our study the inoculation of PGPB affected the expression of both *SlDREB1* and *SlDREB2* in plants subjected to water deficit. Previous analysis on expression of *SlDREB2* in tomato under different stress conditions, including drought, showed high transcript levels only by 6h after the imposed stress, and a rapid declining until 48 h (Guo and Wang, 2011). In our study, a decoupling in the expression of these two genes, belonging to two independent families, was observed in several conditions. It is worth noting that correlation analysis among gene expression data suggested a high positive correlation between *SlDREB1* and five other well-known stress-responsive genes, *i.e.*, *SlHSP20_I, SlHSP20_II, SlJA2, SlNCED1, SlTAS14*, further supporting this hypothesis. Stress responsive elements in plants comprise heat shock proteins. In tomato, it has been proved a regulation of genes coding for heat shock proteins in plants subjected to water deficit (Iovieno et al., 2016). Here, the two assessed heat shock protein related genes, *i.e.*, *SlHSP20_I* and *SlHSP20_II*, showed a contrasting regulation: the one belonging to class II was up-regulated under stress in inoculated plants, while the one belonging to class I was never regulated with the exception of plants inoculated with strain 509 and with combination 509 + 510 under stress. The *HSP20* genes are known to be involved in the response of plants to abiotic stresses such as drought, salt, heat and also play an important role in plant growth and development (Jacob et al., 2017). Yu et al. (2016) found that Solyc09g015000.2 (here corresponding to *SlHSP20_I*) was up-regulated in presence of high temperatures. In our experiment, the gene was up-regulated in stressed plants inoculated with strain 509, while it was down-regulated in stressed plants inoculated with strains 509 + 510, suggesting a primary effect of the inoculation rather than of the stress. In rice, it has been detected the up-regulation of a *HSP20* gene, together with other genes involved in ABA signaling, under drought and in presence of *P. fluorescens*, suggesting a role of the bacterium in inducing the systemic tolerance to drought, regulating the gene expression (Saakre et al., 2017). In addition to ABA metabolism, ethylene pathway is affected by drought in tomato (Pan et al., 2012). Expression of *SlACO4*, a gene with a function in ethylene metabolism, was evaluated. *SlACO4* belongs to a multigene family and is involved in the final step of this pathway, playing a role in the ripening, but also in response to environmental stress (Hamilton et al., 1991; Jia et al., 2018). The down-regulation of this gene was observed in tomato plants particularly under water stress in presence of bacterial strains, suggesting a negative impact on ethylene biosynthesis. Therefore, it has been reported that the negative impact of water deficit in plants can be mitigated through the reduction of ethylene produced by the root system that could then continue to grow maintaining functionality (Brunetti et al., 2021). While in tomato plants the up-regulation of different ACO enzymes in presence of a bacterial inoculation was reported (Ibort et al., 2018), our results agree with Brilli et al. (2019), which observed a down-regulation of ACO-related genes probably due to the effect of *Pseudomonas chlororaphis*. Moreover, pepper plants inoculated with *Bacillus* sp. TW4 and subjected to osmotic stress showed down-regulation of *CaACCO* (Sziderics et al., 2007).

### Synergistic effect of bacterial strains in plant growth and plant response to stress

Although not strong differences have been observed among single and combined treatments, some specific responses can be highlighted such as a synergistic effect between 509 and 510 strains to activate genes putatively playing a role in plant response to stress and to improve plant biomass. As above described, the strain 509 showed the ability to produce IAA, while the 510 exhibited the ability to solubilize iP. The IAA production by the strain 509 may act synergistically by further augmenting plant growth and development, possibly by modulating root architecture, which facilitates a more efficient uptake of nutrients, including P (Duca et al., 2014). Meanwhile, phosphorus solubilization by the strain 510 might enhance the availability of this essential nutrient, fostering an environment conducive for optimal plant growth (Rawat et al., 2021). This synergism can potentially stimulate plant growth, with the IAA production that may promote root elongation and branching, thereby creating a larger root surface area for increased P uptake. Here, the 509 + 510 combination led to a positive trend in plant growth, although the values were not always statistically significantly different from the uninoculated and single inoculated plants. It is worth noting that bacterial species of strains 509 and 510, *i.e.*, *Leucobacter chromiireducens* and *Pseudochrobactrum saccharolyticum*, respectively, are known to be often associated to chromium-contaminated soil (He et al., 2014; Sturm et al., 2018). Despite the initial soil, from which these strains were isolated, was not evaluated for chromium contamination (Sillo et al., 2022), further investigations could be useful to exploit these strains in bio-based products to be potentially used in contaminated agricultural soil.

In conclusion, this study revealed the plant growth promoting potential of different bacterial isolates in tomato plants, both in well-watered and stressed conditions. The used integrated approach allowed to obtain a broader picture of the plant status, from biometric, eco-physiological and molecular point of view. The inoculation with bacterial strains, isolated from soil (509 and 510) and tomato roots (518) determined the regulation of genes involved in pathways related to ABA, MEP, osmoprotectant and heat shock proteins, particularly in plants subjected to water deficit. Although we cannot report data on the plant colonization microscopically showing the presence of endophytic colonization, our study confirms that a synergistic and complementary interaction between diverse PGP bacterial strains is a relevant point to be taken in account for the formulation of inocula.

## Data availability statement

The datasets presented in this study can be found in online repositories. The names of the repository/repositories and accession number(s) can be found below: https://www.ncbi.nlm.nih.gov/, OP364100, OP364101, OP364102, OP364103, OP364104, OP364105, OP364106, OP364107, OP364108, OP364109, OP364110.

## Author contributions

EZ: Investigation, Writing – review and editing, Methodology, Writing – original draft. EF: Conceptualization, Investigation, Methodology, Writing – review and editing. LG: Data curation, Formal Analysis, Investigation, Visualization, Writing – review and editing. FB: Investigation, Writing – review and editing. FS: Data curation, Formal Analysis, Investigation, Visualization, Writing – review and editing. DF: Investigation, Methodology, Writing – review and editing. IP: Investigation, Methodology, Writing – review and editing. MC: Conceptualization, Writing – review and editing. RB: Conceptualization, Investigation, Supervision, Writing – review and editing.

## Supplementary material

Supplementary Figure 1 Pictures of tomato plants of experiment 1 showing the differences in height among treatments at 13/06/2022.

Supplementary Figure 2 Radar plots for each biometric parameters comparing the contribution of each inoculation in WW (grey area) and WS conditions (red line). **(A)** shoot height, **(B)** stem diameter, **(C)** leaf number, **(D)** shoot dry weight, **(E)** root dry weight.

Supplementary Figure 3 Correlation plot among gene expression data. Each diagonal subplot shows the distribution of data of the considered variable as a grey histogram. Scatterplots of each pair of variables with a least-squares reference line (red) are also reported. The slope of red line corresponds to the Pearson correlation coefficient. Numbers in the matrix represent the Pearson correlation coefficients (r). Font size used for correlation coefficients reflects the degree of correlation. Red asterisks showed the *p*-value of the correlation (. = *p* < 0.1, * = *p* < 0.05, ** = *p* < 0.01, *** = *p* < 0.001).

Supplementary Table 1 List of the primers used in RT-qPCR.

Supplementary Table 2 Two-way ANOVA analysis of the biometric parameters and chlorophyll content (CCI).

Supplementary Table 3 Two-way ANOVA analysis of the shoot and root dry weight.

Supplementary Table 4 Fold change with SE of each gene in each treatment in WW condition. The *p*-value is also indicated; those in bold are < 0.05.

Supplementary Table 5 Fold change with SE of each gene in each treatment in WS condition. The *p*-value is also indicated; those in bold are < 0.05.
