# Supplementary figures and images for: Diverse plant promoting bacterial species differentially improve tomato plant fitness under water stress
Source: Front Plant Sci. 2023 Nov 24;14:1297090. doi: 10.3389/fpls.2023.1297090 (PMC10706133; doi:10.3389/fpls.2023.1297090)

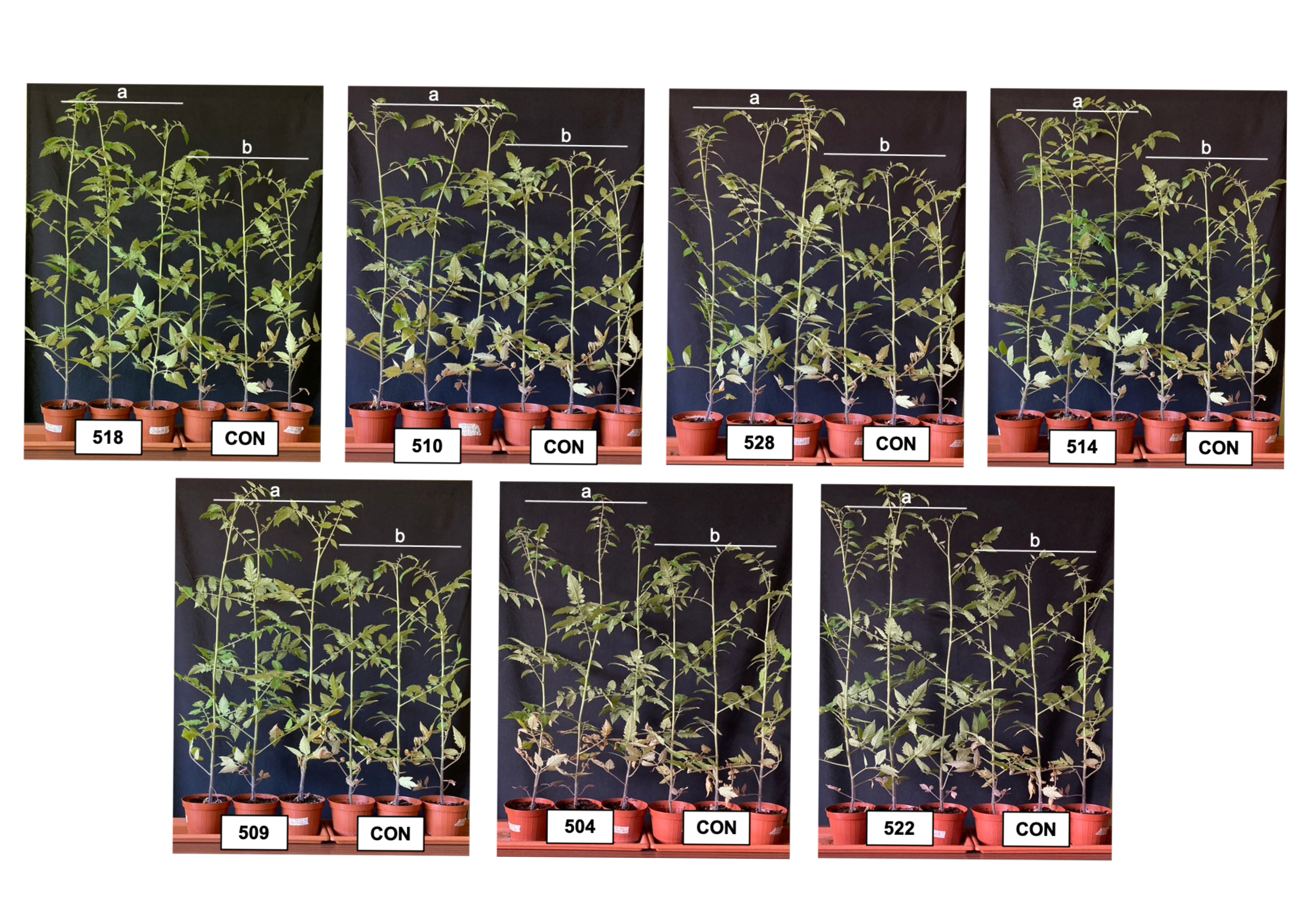

Supplement: Supplementary Figure 1 — Pictures of tomato plants of experiment 1 showing the differences in height among treatments at 13/06/2022. [file Image_1.jpeg]

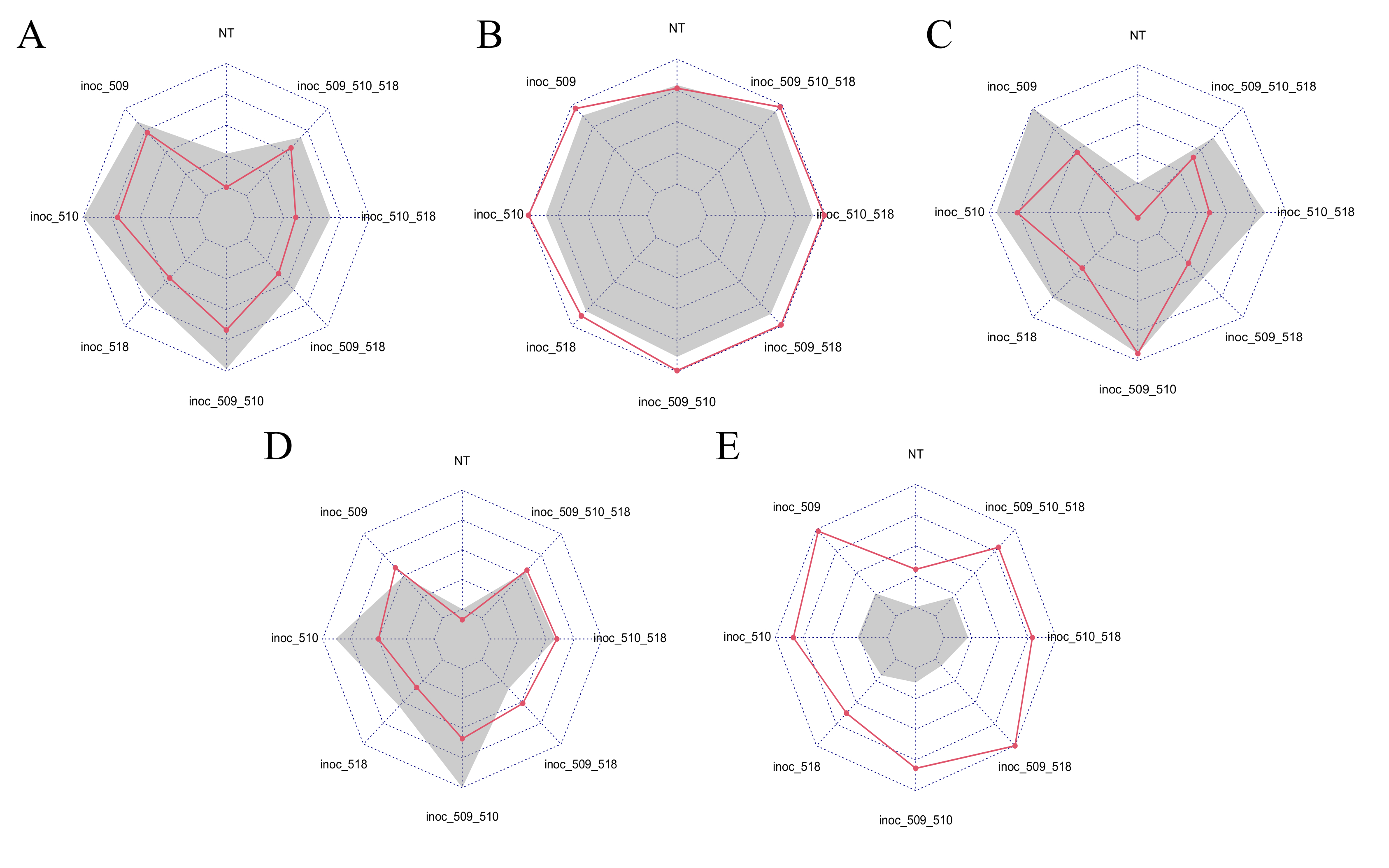

Supplement: Supplementary Figure 2 — Radar plots for each biometric parameters comparing the contribution of each inoculation in WW (grey area) and WS conditions (red line). (A) shoot height, (B) stem diameter, (C) leaf number, (D) shoot dry weight, (E) root dry weight. [file Image_2.jpeg]

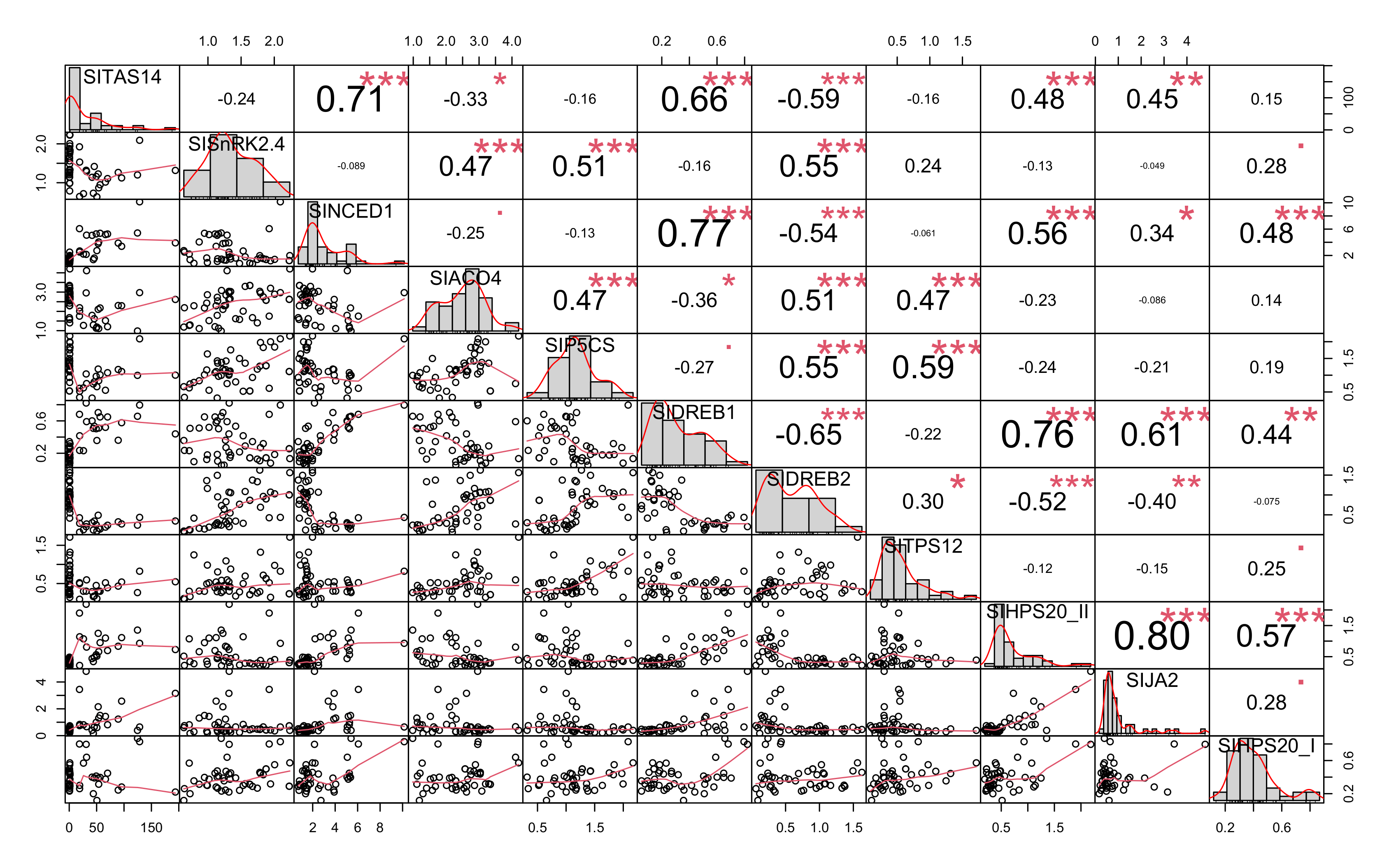

Supplement: Supplementary Figure 3 — Correlation plot among gene expression data. Each diagonal subplot shows the distribution of data of the considered variable as a grey histogram. Scatterplots of each pair of variables with a least-squares reference line (red) are also reported. The slope of red line corresponds to the Pearson correlation coefficient. Numbers in the matrix represent the Pearson correlation coefficients (r). Font size used for correlation coefficients reflects the degree of correlation. Red asterisks showed the p-value of the correlation (. = p < 0.1, * = p < 0.05, ** = p < 0.01, *** = p < 0.001). [file Image_3.jpeg]
