# Supplementary material for: Diverse plant promoting bacterial species differentially improve tomato plant fitness under water stress
Source: Front Plant Sci. 2023 Nov 24;14:1297090. doi: 10.3389/fpls.2023.1297090 (PMC10706133; doi:10.3389/fpls.2023.1297090)
Supplement: Supplementary Table 1 — List of the primers used in RT-qPCR. [file Table_1.docx]

**Supplementary Table S1**. List of the primers used in RT-qPCR.

| **Putative function** | **Gene name** | **Forward primer** | **Reverse Primer** | **Reference** |
| --- | --- | --- | --- | --- |
| Elongation factor 1 alpha | *SlEFα1* | CTCCATTGGGTCGTTTTGCT | GGTCACCTTGGCACCAGTTG | Digilio et al., 2010 |
| Expressed | *Slexpressed* | GCTAAGAACGCTGGACCTAATG | TGGGTGTGCCTTTCTGAATG | Expósito-Rodríguez et al., 2008 |
| Clathrin adaptor complexes medium subunit/endocytic pathway | *SlCAC* | CCTCCGTTGTGATGTAACTGG | ATTGGTGGAAAGTAACATCATCG | Expósito-Rodríguez et al., 2008 |
| 1-aminocyclopropane-1-carboxylic acid oxidase | *SlACO4* | TTCGCGCTCACACGGATGCT | CACCTCTAGCTGATCGCCGAGG | Porcel et al., 2014 |
| 9-cis-epoxycarotenoid dioxygenase | *SlNCED1* | ACCCACGAGTCCAGATTTC | GGTTCAAAAAGAGGGTTAGC | Lopez-Raez et al., 2010 |
| Serine/threonine-protein kinase | *SlSnRK2;4* | TGATAGCAGCGGGGAGATCA | TGCACACCAACAAGTCCAGA | Chitarra et al., 2016 |
| Dehydrin TAS14 | *SlTAS14* | CAATACGGCAATCAAGACCAAA | TGTTTCTTGGACATGGTTTCCA | Chitarra et al., 2016 |
| Pyrroline-5-carboxylate  synthetase | *SlP5CS* | AACTGAGCTTGATGGCAAGG | ACCAGAGGCTGAGCTGATGT | Iovenio et al., 2016 |
| Dehydration responsive element binding protein 1 | *SlDREB1* | CGGATGACCTTGCATTCATAGA | TCTCTTCCTCGCGTCCTATATC | Rai et al., 2019 |
| Dehydration responsive element binding protein 2 (DREB2) | *SlDREB2* | CTTCTTCGTCGCCCTCATC | CCTCTTCACCTCTGCATCTTT | Rai et al., 2019 |
| Terpene synthase | *SlTPS12* | CCCAATGGTTAAACAATGATAATC | ATCATATAGCCAGCACTTACCATC | Falara et al., 2011 |
| Heat shock protein 20 | *SlHSP20_II* | CCGTGGACATTCTGGATACCCCAAA | CCTCTTCTTCACTCTCCTCGCGC | This work |
| Heat shock protein 20 | *SlHSP20_I* | CACCCCATCCTCTGCTCGTGAAA | GAACTTACCGCTGCTCCTCTCCA | This work |
| NAC domain protein | *SlJA2* | TGGGCAATTCTCGCTGGGCT | TCGGAAATTTGGAGGATGGGCGT | This work |
