# Supplementary material for: Diverse plant promoting bacterial species differentially improve tomato plant fitness under water stress
Source: Front Plant Sci. 2023 Nov 24;14:1297090. doi: 10.3389/fpls.2023.1297090 (PMC10706133; doi:10.3389/fpls.2023.1297090)
Supplement: Supplementary Table 2 — Two-way ANOVA analysis of the biometric parameters and chlorophyll content (CCI). [file Table_2.docx]

**Table S2.** Two-way ANOVA analysis of the biometric parameters and chlorophyll content (CCI).

| **Source of Variance** | | **Shoot height (mm)** | | | | **Stem diameter (µm)** | | | **Number of leaves** | | | | **SPAD (CCI)** | | |
| --- | --- | --- | --- | --- | --- | --- | --- | --- | --- | --- | --- | --- | --- | --- | --- |
| **Condition** | *** | | | | *** | | | | *** | | | *** | | | |
| **Inoculation** | *** | | | | *** | | | | *** | | | ns | | | |
| **Condition x Inoculation** | ns | | | | ns | | | | ns | | | ns | | | |
| **Condition** | | |  |  |  | |  |  |  |  |  |  | |  |  |
| WW | 280.65 | | ± | 46.55a | 3349,75 | | ± | 340,16a | 8.60 | ± | 0.77a | 10.55 | | ± | 0.16a |
| WS | 258.43 | | ± | 34.99b | 3629,25 | | ± | 389,17b | 8.13 | ± | 0.74b | 8.69 | | ± | 0.18b |
| **Inoculation** | | |  |  |  | |  |  |  |  |  |  | |  |  |
| NT | 220.42 | | ± | 29.98b | 3044.50 | | ± | 430.37c | 7.15 | ± | 0.49d | 9.24 | | ± | 0.23 |
| 509 | 288.30 | | ± | 38.40a | 3535.00 | | ± | 302.30ab | 8.65 | ± | 0.75abc | 9.61 | | ± | 0.40 |
| 510 | 294.15 | | ± | 38.41a | 3567.00 | | ± | 312.63ab | 8.85 | ± | 0.59ab | 9.51 | | ± | 0.32 |
| 518 | 266.55 | | ± | 30.35a | 3332.5 | | ± | 327.88bc | 8.40 | ± | 0.60bc | 9.66 | | ± | 0.32 |
| 509+510 | 292.00 | | ± | 28.64a | 3599.50 | | ± | 370.72ab | 9.00 | ± | 0.32a | 9.54 | | ± | 0.43 |
| 509+518 | 259.50 | | ± | 46.89ab | 3565.50 | | ± | 395.00ab | 8.15 | ± | 0.75c | 9.63 | | ± | 0.35 |
| 510+518 | 261.25 | | ± | 44.90a | 3619.5 | | ± | 367.30ab | 8.40 | ± | 0.50bc | 9.40 | | ± | 0.41 |
| 509+510+518 | 271.13 | | ± | 34.29a | 3648.5 | | ± | 240.40a | 8.30 | ± | 0.66c | 10.40 | | ± | 0.62 |
| **Condition x Inoculation** | | |  |  |  | |  |  |  |  |  |  | |  |  |
| NT_WW | 238.00 | | ± | 34.73 | 3089.00 | | ± | 500.70 | 7.40 | ± | 0.52 | 9.83 | | ± | 0.34 |
| 509_WW | 294.40 | | ± | 48.98 | 3394.00 | | ± | 183.07 | 9.10 | ± | 0.32 | 10.57 | | ± | 0.60 |
| 510_WW | 308.60 | | ± | 46.94 | 3311.00 | | ± | 183.51 | 9.00 | ± | 0.67 | 10.50 | | ± | 0.37 |
| 518_WW | 277.70 | | ± | 33.90 | 3235.00 | | ± | 360.01 | 8.70 | ± | 0.48 | 10.83 | | ± | 0.20 |
| 509+510_WW | 307.40 | | ± | 25.99 | 3406.00 | | ± | 250.43 | 9.00 | ± | 0.47 | 10.91 | | ± | 0.51 |
| 509+518_WW | 268.70 | | ± | 53.25 | 3349.00 | | ± | 373.67 | 8.30 | ± | 0.95 | 10.47 | | ± | 0.57 |
| 510+518_WW | 276.00 | | ± | 48.06 | 3454.00 | | ± | 364.82 | 8.80 | ± | 0.42 | 10.51 | | ± | 0.58 |
| 509+510+518_WW | 277.00 | | ± | 44.45 | 3560.00 | | ± | 272.07 | 8.50 | ± | 0.71 | 10.77 | | ± | 0.43 |
| NT_WS | 211.50 | | ± | 29.58 | 3000.00 | | ± | 368.66 | 6.90 | ± | 0.32 | 8.64 | | ± | 0.20 |
| 509_WS | 282.20 | | ± | 25.11 | 3676.00 | | ± | 339.45 | 8.20 | ± | 0.79 | 8.65 | | ± | 0.35 |
| 510_WS | 279.70 | | ± | 21.17 | 3823.00 | | ± | 164.39 | 8.70 | ± | 0.48 | 8.52 | | ± | 0.27 |
| 518_WS | 255.40 | | ± | 22.78 | 3430.00 | | ± | 276.08 | 8.10 | ± | 0.57 | 8.49 | | ± | 0.31 |
| 509+510_WS | 276.60 | | ± | 23.00 | 3793.00 | | ± | 379.77 | 9.00 | ± | 0.00 | 8.17 | | ± | 0.31 |
| 509+518_WS | 250.30 | | ± | 40.23 | 3782.00 | | ± | 292.57 | 8.00 | ± | 0.47 | 8.78 | | ± | 0.22 |
| 510+518_WS | 246.50 | | ± | 38.24 | 3785.00 | | ± | 301.41 | 8.00 | ± | 0.00 | 8.28 | | ± | 0.31 |
| 509+510+518_WS | 265.20 | | ± | 20.70 | 3737.00 | | ± | 174.87 | 8.10 | ± | 0.57 | 10.02 | | ± | 1.19 |

All biometric data are expressed as mean ± SD, while chlorophyll content as mean ± SE. ns, *, **, ***: not significant or significant at *p* ≤ 0.05, *p* ≤ 0.01 and *p* ≤ 0.001, respectively. Different letters within each column indicate significant differences according to Tukey HSD test (*p* ≤ 0.05). WW: well-watered. WS: water stress.
