# Supplementary material for: Diverse plant promoting bacterial species differentially improve tomato plant fitness under water stress
Source: Front Plant Sci. 2023 Nov 24;14:1297090. doi: 10.3389/fpls.2023.1297090 (PMC10706133; doi:10.3389/fpls.2023.1297090)
Supplement: Supplementary Table 3 — Two-way ANOVA analysis of the shoot and root dry weight. [file Table_3.docx]

**Table S3.** Two-way ANOVA analysis of the shoot and root dry weight.

| **Source of Variance** | **Shoot dry weight (mg)** | | | **Root dry weight (mg)** | | |
| --- | --- | --- | --- | --- | --- | --- |
| **Condition** | ns | | | *** | | |
| **Inoculation** | *** | | | ** | | |
| **Condition * Inoculation** | ns | | | ns | | |
| **Condition** | |  |  |  |  |  |
| WW | 806.50 | ± | 189.81 | 187.25 | ± | 71.47b |
| WS | 757.50 | ± | 158.51 | 465.00 | ± | 154.51a |
| **Inoculation** | |  |  |  |  |  |
| NT | 512.00 | ± | 82.44b | 181.00 | ± | 82.25b |
| 509 | 805.00 | ± | 127.48a | 396.00 | ± | 236.84a |
| 510 | 877.00 | ± | 179.07a | 366.00 | ± | 171.09a |
| 518 | 751.00 | ± | 140.51a | 297.00 | ± | 139.53ab |
| 509+510 | 928.00 | ± | 141.88a | 326.00 | ± | 185.06ab |
| 509+518 | 746.00 | ± | 156.15a | 358.00 | ± | 236.21a |
| 510+518 | 826.00 | ± | 143.70a | 342.00 | ± | 189.26a |
| 509+510+518 | 811.00 | ± | 97.12a | 343.00 | ± | 162.14a |
| **Condition * Inoculation** | |  |  |  |  |  |
| NT_WW | 534.00 | ± | 112.61 | 110.00 | ± | 48.48 |
| 509_WW | 780.00 | ± | 140.89 | 230.00 | ± | 139.82 |
| 510_WW | 972.00 | ± | 139.71 | 234.00 | ± | 41.59 |
| 518_WW | 806.00 | ± | 106.21 | 196.00 | ± | 47.22 |
| 509+510_WW | 1030.00 | ± | 102.97 | 164.00 | ± | 19.49 |
| 509+518_WW | 702.00 | ± | 168.88 | 144.00 | ± | 49.30 |
| 510+518_WW | 822.00 | ± | 175.41 | 210.00 | ± | 62.05 |
| 509+510+518_WW | 806.00 | ± | 117.18 | 210.00 | ± | 20.00 |
| NT_WS | 490.00 | ± | 37.42 | 252.00 | ± | 16.43 |
| 509_WS | 830.00 | ± | 123.09 | 562.00 | ± | 194.35 |
| 510_WS | 782.00 | ± | 173.41 | 498.00 | ± | 143.42 |
| 518_WS | 696.00 | ± | 159.94 | 398.00 | ± | 126.77 |
| 509+510_WS | 826.00 | ± | 93.17 | 488.00 | ± | 105.21 |
| 509+518_WS | 790.00 | ± | 146.63 | 572.00 | ± | 92.84 |
| 510+518_WS | 830.00 | ± | 125.10 | 474.00 | ± | 182.15 |
| 509+510+518_WS | 816.00 | ± | 86.20 | 476.00 | ± | 120.54 |

All biometric data are expressed as mean ± SD. ns, *, **, ***: not significant or significant at *p* ≤ 0.05, *p* ≤ 0.01 and *p* ≤ 0.001, respectively. Different letters within each column indicate significant differences according to Tukey HSD test (*p* ≤ 0.05). WW: well-watered. WS: water stress.
