# Supplementary material for: Diverse plant promoting bacterial species differentially improve tomato plant fitness under water stress
Source: Front Plant Sci. 2023 Nov 24;14:1297090. doi: 10.3389/fpls.2023.1297090 (PMC10706133; doi:10.3389/fpls.2023.1297090)
Supplement: Supplementary Table 5 — Fold change with SE of each gene in each treatment in WS condition. The p-value is also indicated; those in bold are < 0.05. [file DataSheet_1.docx]

Supplementary Material

# Supplementary Data

At the end of the experiment, DNA was isolated from the roots of inoculated tomatoes (five replicates) with strains 518, 509 and 510, and one plant randomly chosen among those uninoculated ones, using DNeasy plant mini kit (Qiagen SA, Courtaboeuf, France) and following the manufacturer’s instructions. 16S rRNA gene sequence was amplified with primers 27F (5’ GAGAGTTTGATCCTGGCTCAG 3’) (Melničáková et al., 2013) and 1495R (5’ CTACGGCTACCTTGTTACGA 3’) (Ventura et al., 2002), using the Hot Start PCR Master Mix (Thermo Fisher Scientific, Waltham, US). PCR setting for 27-1495 amplification was the following: 2 min at 95°C; 30 s at 94°C, 30 s at 55°C, 90 s at 72°C (34 cycles); 7 min at 72°C. The PCR products were checked on agarose gel (0.8%). On the bases of 16S sequence a primer pair for strains 518, 509 and 510 was designed (518f CCGGATTTATTGGTTTAAAGGGT and 518r CTCCTTGCGGTCACATGC; 509f AAGAGTGGCGAAGGGGTG and 509r AGAGATCTGCCTTCGCCATC; 510f TCTCTACGGACTAACTCGGGA and 510 r AGGGTATCTAATCCTGTTGGCT) using Primer3 (https://primer3.ut.ee/). The PCR mix was the same used for the universal primers, before described, adding 1 ul (50 µM) of PNA for mitochondrion (GGCAAGTGTTCTTCGGA) and plastidial (GGCTCAACCCTGGACAG) DNA in each tube. PCR setting was the same used for the PCR described above except for the annealing temperature (Ta) that was 57.9°C, 63°C and 50°C, respectively for 518, 509 and 510 strain. The PCR products were checked on agarose gel (0.8%), then purified using QIAquick PCR Purification Kit (Qiagen) and sequenced by LMU sequencing (Germany).

The DNA of each sample was successfully amplified with the universal primers for 16S. The DNA extraction, PCR with specific primers for strain 518, amplicon visualization and sequencing confirmed that four out of five plants inoculated with strain 518 contained the bacterial DNA. The amplification with primers for strain 509 yielded results that were not exclusively specific, as they also amplified other bacterial species present in the root DNA. In addition, primers designed for strain 510 failed in specifically detecting DNA from inoculated samples.

# Supplementary Material References (only in Table S1 and Supplementary data)

Digilio, M.C., Corrado, G., Sasso, R., Coppola, V., Iodice, L., Pasquariello, M., et al. (2010). Molecular and Chemical Mechanisms Involved In Aphid Resistance In Cultivated Tomato. New Phytol. 187:1089-1101. doi: 10.1111/j.1469-8137.2010.03314.x

Melničáková, J., Derdáková, M., and Barák, I.A. (2013). System to Simultaneously Detect Tick-Borne Pathogens Based on the Variability of the 16S Ribosomal Genes. Parasit. Vectors 6:269. doi: 10.1186/1756-3305-6-269

Ventura, M.; and Zink, R. (2002). Specific Identification and Molecular Typing Analysis of *Lactobacillus johnsonii* by Using PCR-Based Methods and Pulsed-Field Gel Electrophoresis. FEMS Microbiol. Lett. 217:141-154. doi:10.1111/j.1574-6968.2002.tb11468.x
